# Pharmacological Modulation of Nrf2/HO-1 Signaling Pathway as a Therapeutic Target of Parkinson’s Disease

**DOI:** 10.3389/fphar.2021.757161

**Published:** 2021-11-23

**Authors:** Yumin Wang, Luyan Gao, Jichao Chen, Qiang Li, Liang Huo, Yanchao Wang, Hongquan Wang, Jichen Du

**Affiliations:** ^1^ Department of Respiratory and Critical Care Medicine, Aerospace Center Hospital, Peking University Aerospace School of Clinical Medicine, Beijing, China; ^2^ Department of Neurology, Tianjin Fourth Central Hospital, The Fourth Central Hospital Affiliated to Nankai University, The Fourth Central Clinical College, Tianjin Medical University, Tianjin, China; ^3^ Department of Neurology, The Affiliated Hospital of Chifeng University, Chifeng, China; ^4^ Department of Pediatric Neurology, Shengjing Hospital of China Medical University, Shenyang, China; ^5^ Department of Neurology, Aerospace Center Hospital, Peking University Aerospace School of Clinical Medicine, Beijing, China

**Keywords:** Parkinson’s disease, oxidative stress, Nrf2, heme oxygenase-1, neuroprotection

## Abstract

Parkinson’s disease (PD) is a complex neurodegenerative disorder featuring both motor and nonmotor symptoms associated with a progressive loss of dopaminergic neurons in the substantia nigra pars compacta. Oxidative stress (OS) has been implicated in the pathogenesis of PD. Genetic and environmental factors can produce OS, which has been implicated as a core contributor to the initiation and progression of PD through the degeneration of dopaminergic neurons. The transcription factor nuclear factor erythroid 2-related factor 2 (Nrf2) orchestrates activation of multiple protective genes, including heme oxygenase-1 (HO-1), which protects cells from OS. Nrf2 has also been shown to exert anti-inflammatory effects and modulate both mitochondrial function and biogenesis. Recently, a series of studies have reported that different bioactive compounds were shown to be able to activate Nrf2/antioxidant response element (ARE) and can ameliorate PD-associated neurotoxin, both in animal models and in tissue culture. In this review, we briefly overview the sources of OS and the association between OS and the pathogenesis of PD. Then, we provided a concise overview of Nrf2/ARE pathway and delineated the role played by activation of Nrf2/HO-1 in PD. At last, we expand our discussion to the neuroprotective effects of pharmacological modulation of Nrf2/HO-1 by bioactive compounds and the potential application of Nrf2 activators for the treatment of PD. This review suggests that pharmacological modulation of Nrf2/HO-1 signaling pathway by bioactive compounds is a therapeutic target of PD.

## Introduction

Parkinson’s disease (PD) is defined primarily as a movement disorder, with the typical symptoms being resting tremor, rigidity, bradykinesia, and postural instability (Rai et al., 2020; Rai and Singh, 2020; Rai et al., 2021). PD is pathologically characterized by degeneration of nigrostriatal dopaminergic neurons and the presence of Lewy bodies (LBs), which mainly consist of misfolded α-synuclein, ubiquitin, Parkin, PTEN-induced kinase-1 (PINK1), and other proteins in the surviving neurons (Rai et al., 2017, 2019a; Zahra et al., 2020; Oliveira et al., 2021). PD is the second most common age-related neurodegenerative disease, affecting more than 2% of the population older than 65 years old (Aarsland et al., 2017). PD is becoming a major age-related health problem (Zou Y. et al., 2015; Hirsch et al., 2016; Savica et al., 2016).

The majority of PD cases are idiopathic or sporadic, and approximately 10% of PD cases are associated with a genetic component. Even though familial PD is the less frequent form as only 10% of cases comprise only a minor subset of the overall PD pool, these are of high relevance since they have provided extended information about pathogenesis (Cuenca et al., 2018; Deng et al., 2018; Domingo and Klein, 2018). Since the first PD-associated substitution mutation of alanine in position 53 for threonine (A53T) in α-synuclein was identified more than 20 years ago (Polymeropoulos et al., 1997), many other genes with Mendelian inheritance have been identified, and the number of PD-related genes as risk factors has exponentially increased (Brás and Outeiro, 2021; Oliveira et al., 2021). Twenty-three loci and nineteen genes have been directly linked to the cause of genetic PD (Deng et al., 2018) (Table 1). PINK1, leucine-rich repeat kinase 2 (LRRK2), Parkin, DJ-1, and α-synuclein are the proteins that have been strongly linked to the familial PD (Polymeropoulos et al., 1997; Bonifati et al., 2003; Valente et al., 2004; Di Fonzo et al., 2005; Nichols et al., 2005). Of note, because of its predominance in LBs, α-synuclein is most commonly associated with PD pathogenesis (Spillantini et al., 1997). These different mutation genes are involved in the regulation of different pathways, Parkin, and UCHL-1 for proteasomal degradation pathways; PINK1, Omi/Htra, DJ-1, and LRRK2 for mitochondrial homeostasis; DJ-1 for antioxidant response pathways; ATP13A2 for lysosome function; and PINK1 and Parkin for mitophagy.

**TABLE 1:** Common monogenic forms of Parkinson’s disease-causing locus and genes.

References	Locus name	Locus location	Gene name	Symbol	Clinical features	LBs	Inheritance	Pathogenic mutation(s)
Polymeropoulos et al. (1997); Farrer et al. (1999)	PARK1PARK4	4q22.1	α-Synuclein	SNCA	EO(PARK4); LO	Yes	AD	MUs (A53T, A30P, A18T, A29S, E46K, H50Q, G51D, and A53E); multiplications (duplications and triplications)
Paisán-Ruíz et al. (2004)	PARK8	12q12	Leucine-rich repeat kinase 2 gene	LRRK2	LO	Yes	AD	MUs [I1371V, N1437H, R1441C, R 1441G, R1441H, Y1699C, G2019S (most common), and I2020T]
Vilariño-Güell et al. (2011)	PARK17	16q11.2	Vacuolar protein sorting 35	VPS35	LO	No	AD	MU (D620N)
Kitada et al. (1998)	PARK2	6q26	Parkin	PRKN	EO	No	AR	ERs, including exon deletions or multiplications (most common); MUs and NMs, small deletions or insertions; splice-site alterations
Valente et al. (2001)	PARK6	1p36	PTEN-induced kinase-1	PINK1	EO	No	AR	MUs or NMs (most common); ERs, including exon deletions or duplications
van Duijn et al. (2001)	PARK7	1p36.23	Parkinsonism-associated deglycase gene	DJ-1	EO	No	AR	MUs or ERs (most common); splice-site alterations

AD, autosomal dominant; AR, autosomal recessive; EO, early onset; LO, late onset; MUs, missense mutations; NMs, nonsense mutations; ERs, exon rearrangements.

Despite all the efforts that have been directed to interpret which mechanisms are responsible for neuronal degeneration in PD, its origin and the cause of PD remain unknown in most patients and remain to be fully elucidated (Przedborski, 2017), leading to unsustainable treatment options that only provide symptomatic relief, and there are no preventative or curative therapies that slow the neurodegenerative process. Most PD cases have a multifactorial etiology and a complicated interplay of genetic and environmental factors, which affect numerous fundamental cellular processes. Since 1992, the oxidative stress (OS) hypothesis came into existence with an observation of postmortem brain of PD patients (Fahn and Cohen, 1992); accumulating evidence indicates that OS leads to the neurodegeneration of these DA neurons (Dias et al., 2013; Blesa et al., 2015; Sarrafchi et al., 2016; Puspita et al., 2017; Guo et al., 2018). It is now believed that OS plays an important role during the pathogenesis of PD (Subramaniam and Chesselet, 2013). Ample evidence has supported the OS hypothesis, which prompted an investigation into the efficacy of nonenzymatic exogenous antioxidants to treat PD (Todorovic et al., 2016). More recently, much attention and interest have been centered on targeting antioxidant gene transcription through pharmacological modulation, which leads to mitigating OS-dependent neuronal injury (Buendia et al., 2016). The common target is Nrf2, which is a transcription factor and “master regulator.” Cells have been equipped with a complex endogenous protection system against OS through the antioxidant response element (ARE) pathway, which renders neuronal cells resistant to OS. The nuclear factor E2-related factor 2 (Nrf2) regulates this coordinated induction of detoxifying and antioxidative enzymes through the binding of the ARE within the regulatory region of target genes. Nrf2 regulates the coordinated expression of cytoprotective genes, including heme oxygenase-1 (HO-1), among other enzymes (Jiang L. et al., 2016; Deshmukh et al., 2017). Thus, considering the neuroprotective role of the Nrf2/HO-1 pathway, pharmacological modulation of the activation of Nrf2/HO-1 may represent a novel therapeutic target for the treatments of PD (Cuadrado et al., 2018). Currently, ongoing investigations have been focused on the potential of natural compounds targeting the Nrf2/HO-1 signaling pathway as a neuroprotective agent for the therapeutic treatment of PD. Therefore, it will be vital to summarize the current literature on Nrf2/HO-1 signaling pathway in PD.

Here, we briefly overview the sources of OS and the association between OS and the pathogenesis of PD. Then, we provided a concise overview of the Keap1/Nrf2/ARE pathway and delineated the role played by activation of Nrf2/HO-1 in PD. Following this background, we expand our discussion to the neuroprotective effects of pharmacological modulation of Nrf2/HO-1 by bioactive compounds and the potential application of Nrf2 activators for the treatment of PD. This review suggests that pharmacological modulation of Nrf2/HO-1 signaling pathway by bioactive compounds is a therapeutic target of PD.

## The Role of Oxidative Stress in Parkinson’s Disease

### General Aspects Regarding Oxidative Stress

OS was first introduced by Helmut Sies in 1985, who stated “A disturbance in the prooxidant/antioxidant systems in favor of the former may be denoted as an OS” (Sies, 2020a; Lushchak and Storey, 2021). More recently, OS was defined as a disequilibrium between the levels of produced reactive oxygen species (ROS) and the ability of a biological system to readily detoxify the reactive intermediates or to repair the resulting damage, creating a perilous state contributing to cellular damage (Ji and Yeo, 2021). Many complex mechanisms maintained the delicate balance between ROS generation and elimination. The dysfunction of any of these mechanisms could result in alterations in cellular redox status. An increase in ROS production or a decrease in ROS-scavenging capacity resulting from exogenous stimuli or endogenous metabolic alterations can disrupt redox homeostasis, leading to OS.

ROS is a collective term that describes the oxygen-derived small molecules that are formed upon incomplete reduction of oxygen. ROS includes oxygen radicals and certain nonradicals that either are oxidizing agents or are easily converted into radicals. Oxygen radicals include O_2_^•–^ (superoxide anion), HO^•^ (hydroxyl radical), RO_2_^•^ (peroxyl), and RO^•^ (alkoxyl), and certain nonradicals include HOCl (hypochlorous acid), O_3_ (ozone),^1^O_2_ (singlet oxygen), and H_2_O_2_ (hydrogen peroxide) (Bedard and Krause, 2007; D'Autréaux and Toledano, 2007). The greater chemical reactivity of ROS with regard to oxygen mediates the toxicity of oxygen (Gutowski and Kowalczyk, 2013).

O_2_^•–^ is considered the “primary” ROS, which is produced mainly by mitochondrial complexes I and III of the electron transport chain (ETC), is highly reactive, and can easily cross the inner mitochondrial membrane (IMM), where it can be reduced to H_2_O_2_ (Elfawy and Das, 2019).

O_2_^•–^ can further interact with other molecules to generate “secondary” ROS either directly or prevalently through enzyme- or metal-catalyzed processes. The “secondary” ROS are highly reactive and can attack and damage DNA, purines, pyrimidines, deoxyribose backbone, leading to mutation (Pisoschi et al., 2021). OS causes injury to macromolecular components (DNA, proteins, and lipids), which lead to various pathological conditions and human diseases, such as PD.

ROS can be either harmful or beneficial to living systems, which make them play a dual role as both deleterious and beneficial species. ROS exerts beneficial effects at low to moderate concentrations, which involve physiological roles in cellular responses to noxia, such as in defense against infectious agents and cellular signaling systems (Sachdev et al., 2021). The balance between harmful and beneficial effects of free radicals is a very important aspect of living organisms. This balance is achieved by mechanisms called “redox regulation.” The process of “redox regulation” maintains “redox homeostasis” and protects living organisms from various OS and by controlling the redox status *in vivo* (Sies, 2020b).

In response to OS, cells have developed and are equipped with an antioxidant defense system, which uses enzymatic and nonenzymatic antioxidant systems to eliminate ROS and maintain redox homeostasis, thereby protecting cells from damage (Trachootham et al., 2008). Nonenzymatic defenses are the thiol-containing small molecules, including compounds of intrinsic antioxidant properties, such as thioredoxin (Txn), glutathione (GSH), vitamins C and E, and β-carotene. Purely enzymatic defenses ROS-inactivating enzymes, such as glutathione peroxide (GPx), superoxide dismutases (SOD), catalases (CAT), and peroxidases, can exert a protective effect through directly scavenging superoxide radicals and hydrogen peroxide, therefore converting them to less reactive species (Jung and Kwak, 2010). CAT, SOD, and GPx directly neutralize ROS. GSH and Txn neutralize ROS *via* direct interactions serving as substrates for GPx and peroxiredoxins (Prxs). CAT, GPx, and Prxs reduce hydrogen peroxide to water. Antioxidants can be classified into endogenous and exogenous or direct antioxidants, indirect antioxidants, and bifunctional antioxidants according to source, nature, and mechanism of action (Dinkova-Kostova and Talalay, 2008; Magesh et al., 2012). Direct antioxidants are redox-active and short-lived, and they are consumed during the process and need to be regenerated to offer further protection. Indirect antioxidants show with or without redox activity and exert their antioxidant effects through upregulating various antioxidant genes such as HO-1, NAD(P)H, NAD(P)H:quinone oxidoreductase 1 (NQO1), glutathione S-transferase (GST), glutamate-cysteine ligase (GCL), SOD, GPx, CAT, and Txn (Talalay, 2000). These protective proteins have relatively long half-lives, are not consumed during their antioxidant actions, are members of this antioxidant system, and are referred to as the “ultimate antioxidants.” They catalyze various chemical detoxification reactions related to the regeneration of some direct antioxidants (Dinkova-Kostova and Talalay, 2008; Magesh et al., 2012). The Keap1, Nrf2, and ARE are the three main cellular components involved in regulating antioxidant response. The Keap1/Nrf2/ARE is a major signaling pathway that regulates the basal and inducible expression of a wide array of antioxidant genes (Cuadrado et al., 2019). The Keap1/Nrf2/ARE signaling pathway induces an adaptive response for OS that can otherwise lead to PD. Thus, targeting the Keap1/Nrf2/ARE pathway is being regarded as a rational strategy to prevent and treat PD.

### Evidence of Oxidative Stress in PD

OS leads to cellular dysfunction and eventual cell death in both familial and sporadic forms of PD. Both postmortem studies, modeling of PD in animals with toxins in neuronal degeneration of the DAergic nigral neurons (Gilgun-Sherki et al., 2001; Mythri et al., 2011), and *in vivo* observations of patients with PD supported the occurrence of OS in PD (Vinish et al., 2011).

Ample of studies on postmortem brain tissues of PD patients has shown decreased levels of antioxidant enzyme activity (including GPx and CAT), reduced levels of GSH, elevated free iron levels, an augmented activity of SOD, and a decreased mitochondrial complex I activity in the SN of PD patients (Morris and Edwardson, 1994; Pearce et al., 1997; Blum et al., 2001; Jenner, 2003). Evidence showed a selective loss of GSH in the SN (Sian et al., 1994), which is thought of to be one of the earliest biochemical changes in PD (Perry et al., 1982, 1984; Riederer et al., 1989; Danielson and Andersen, 2008) and is not found in other parts of the brain (Sian et al., 1994). Studies have also demonstrated a reduction in mitochondrial complex I activity in PD compared to controls (Mizuno et al., 1989; Parker et al., 1989; Schapira et al., 1989; Mann et al., 1992).

Accumulating evidence indicates that OS markers, such as high levels of oxidatively modified lipids, proteins, and DNA/RNA, are all found in the SNpc of postmortem brains of PD patients. Compared with other brain regions and age-matched controls, cholesterol lipid hydroperoxides and malondialdehyde, the lipid peroxidation products, are 10-fold higher (Dexter et al., 1989). The amounts of nitrotyrosine (3-NT), a marker of damage to protein, have been identified in peripheral polymorphonuclear cells in PD patients and increased in their brains in LBs (Good et al., 1998; Gatto et al., 2000). Increased levels of carbonyl modifications of soluble proteins are also found throughout the brain in PD (Alam et al., 1997a). Meanwhile, the byproduct of lipid peroxidation, 4-hydroxyl-2-nonenal (HNE), is also increased in the SN of PD patients (Yoritaka et al., 1996). Lastly, DNA and RNA oxidation products 8-hydroxydeoxyguanosine (8-OHdG) and 8-hydroxy-guanosine (8-OHG) are also increased in the SN and cerebrospinal fluid of PD patients (Alam et al., 1997b; Zhang et al., 1999; Kikuchi et al., 2002; Isobe et al., 2010).

Evidence of OS existing in PD is further supported by PD animals modeled with toxins that can cause OS, which includes 1-methyl-4-phenyl-1,2,3,6-tetrahydropyridine (MPTP) (Dong et al., 2021), rotenone (Sharma et al., 2021), paraquat (Ahmad et al., 2021), and 6-hydroxydopamine (6-OHDA) (Zou et al., 2021). Moreover, *in vivo* observations revealed that several markers of OS are altered in the cerebrospinal fluid and blood samples of PD patients (Vinish et al., 2011).

## The Sources of Oxidative Stress in Parkinson’s Disease

**FIGURE 1:**
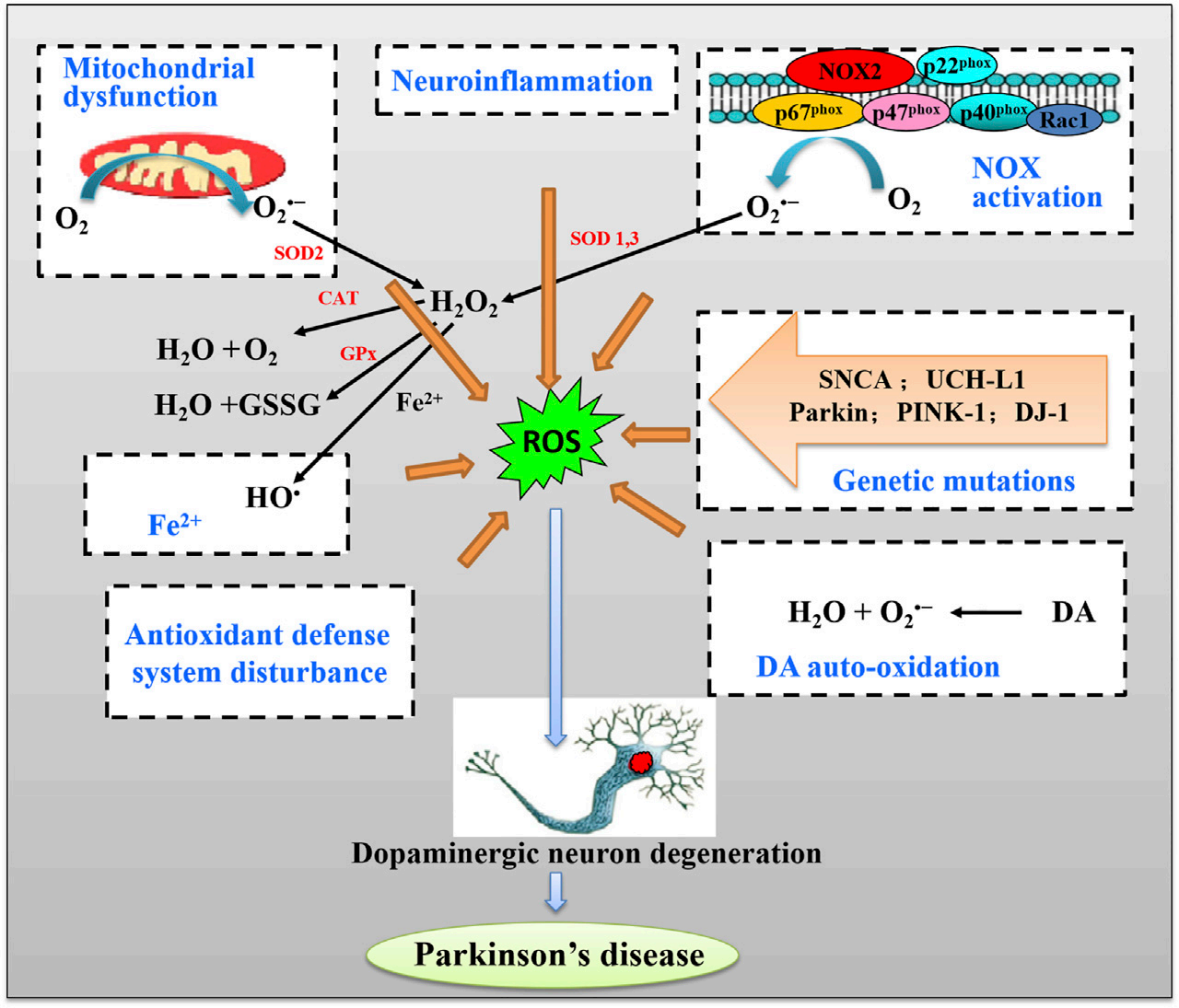
The schematic pathway of major sources of oxidative stress (OS) and induction of DA neuron death in Parkinson’s disease (PD).

Numerous pieces of evidence suggest that a number of sources and mechanisms for OS are recognized in PD. The major sources of OS in PD include mitochondrial dysfunction, nicotinamide adenine dinucleotide phosphate (NADPH) oxidases (NOX) activation, the metabolism of dopamine by autooxidation, and iron (Fe^2+^) accumulation (Figure 1). We will discuss how these PD-associated factors induce ROS and how ROS results in cell death in dopaminergic neurons in PD.

### Mitochondrial Dysfunction and Oxidative Stress in PD

**FIGURE 2:**
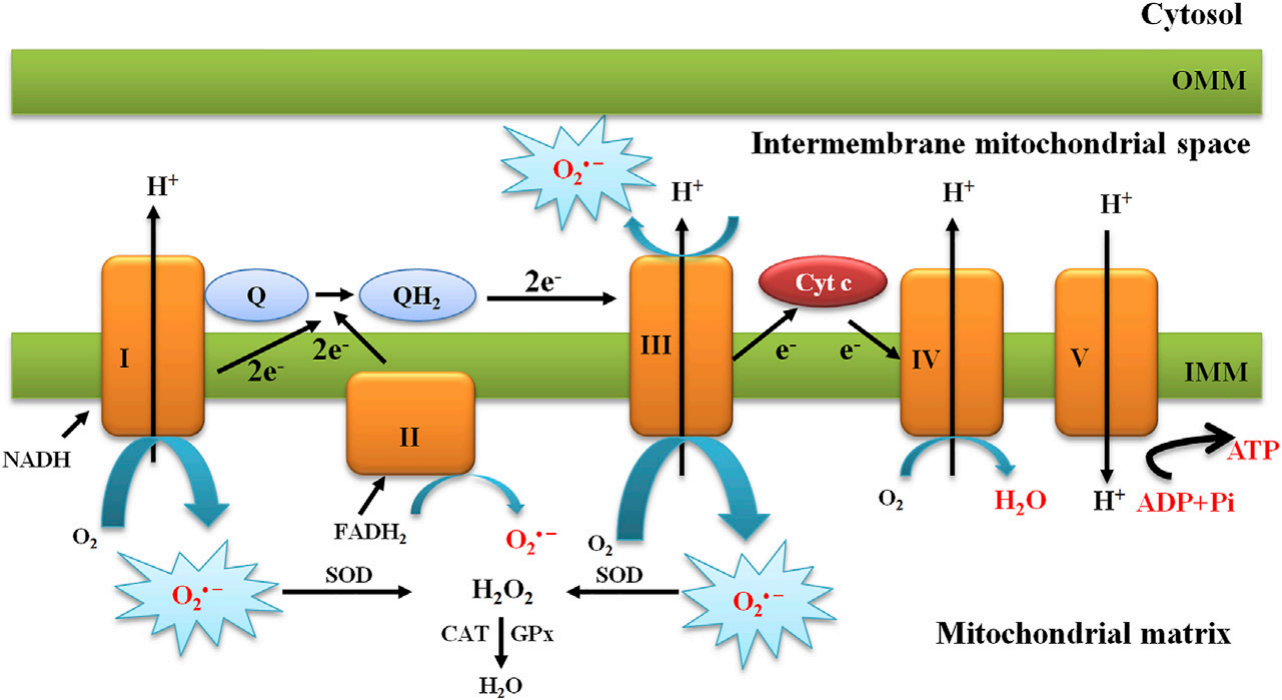
Schematic presentation of the mitochondrial electron transport chain and production of mitochondrial O_2_^•−^. The mitochondrial electron transport chain produces ROS. Mitochondrial complexes I and II use electrons donated from NADH and FADH_2_ to reduce coenzyme Q. Coenzyme Q shuttles these electrons to complex III, where they are transferred to cytochrome c. Complex IV uses electrons from cytochrome c to reduce molecular oxygen to water. The action of complexes I, III, and IV produce a proton electrochemical potential gradient, the free energy of which is used to phosphorylate ADP at ATP synthase. Complexes I, II, and III produce superoxide through the incomplete reduction of oxygen to superoxide, whereas complexes I and II produce superoxide only into the mitochondrial matrix and complex III produces superoxide into both the matrix and the intermembrane space.

Mitochondria are an organelle for their cellular function essential for their role in ATP production, calcium homeostasis, and apoptotic signaling. In eukaryotic cells, mitochondria are the primary source of energy through the process of respiration and oxidative phosphorylation (OXPH) to produce adenosine triphosphate (ATP). The process of OXPH involves coupling of both redox and phosphorylation reactions in the IMM, leading to effective ATP synthesis. During this process, electrons donated from nicotinamide adenine dinucleotide (NADH) or flavin adenine dinucleotide (FADH_2_) are transported through the ETC, which is comprised of complexes I–IV, to produce water and create a proton electrochemical gradient across the IMM (Subramaniam and Chesselet, 2013). The ETC constitutes electron carriers that transport electrons from reduced cofactors, which are reduced during the catabolism of energy nutrients, to molecular oxygen. This comprises the primary energy transformation step. The designated protonmotive force, i.e., the dual gradient across the IMM, is composed of a pH and electrical potential, which provides the driving force for ATP synthesis through the backflow of protons into the mitochondrial matrix through the ATP synthase complex (Figure 2). Protons flow back into the mitochondrial matrix providing energy for the ATP synthase to phosphorylate ADP into ATP. This metabolic process is a critical means of energy production and the main source of O_2_^•–^ and H_2_O_2_ as a major byproduct, leading to propagation of free radicals, thereby contributing to the disease (Boveris and Chance, 1973; Turrens, 2003; Figueira et al., 2013) (Figure 2).

The main sites of ROS production in mitochondria are considered to be complexes I III in the ETC. The primary ROS produced in mitochondria is O_2_^•–^, which results from a single electron transfer to O_2_ in the respiratory chain. Superoxide dismutase 2 (SOD2) or MnSOD converts O_2_^•–^ to H_2_O_2_, which is further detoxified by the CAT. Redox-active metals such as Fe^2+^ also contribute to ROS generation. The highly reactive HO^•^ can be generated through the Fenton reaction or Haber-Weiss reaction in the presence of Fe^2+^, causing severe oxidative damage to the cellular components and leading to DNA damage and lipid damage (Kehrer, 2000) (Figure 2).

**FIGURE 3:**
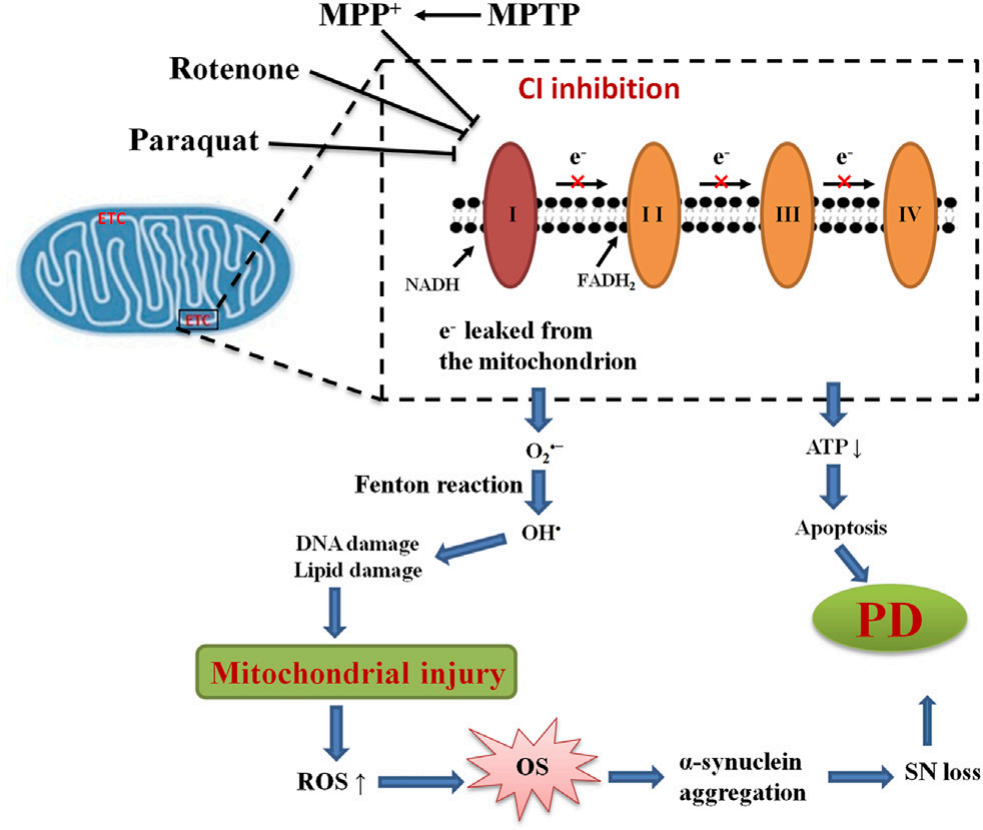
Mitochondrial dysfunction and OS in PD. The mechanism of mitochondrial dysfunction and OS highlights the inhibition of mitochondrial complex I (CI) and associated ROS production leading to loss of dopaminergic neurons in PD.

Many lines of evidence provide substantial evidence that mitochondrial dysfunction involves in the pathogenesis of PD. Histology of postmortem brains of PD patients, which supports the notion of mitochondrial dysfunction, is a common pathological mechanism employed in PD pathology (Chaturvedi and Beal, 2008; Isobe et al., 2010). Accidental administration of MPTP in young drug users, who eventually developed parkinsonism, reveals significant lesions of DAergic neurons in the SNpc (Langston et al., 1983). It was reported that deficiency in mitochondrial complex I was identified for the first time in PD brains but remains normal in other neuronal regions (Schapira et al., 1989, 1990). Since then, ample evidence has been well documented on the role of mitochondrial dysfunction in the pathogenesis of PD. Mounting evidence has shown that mitochondrial dysfunction is one unique feature observed in PD (Figure 3) (Prasuhn et al., 2020). Numerous studies have suggested that mitochondria are the primary source of ROS and contribute to the intracellular OS in PD (Onyango, 2008; Hauser and Hastings, 2013; Subramaniam and Chesselet, 2013). Complex I deficiencies of the ETC account for the majority of sources of ROS in PD. Premature electron leakage from complex I and complex III of ETC to oxygen is the main source of mitochondrial O_2_^•–^(Kussmaul and Hirst, 2006). The dysfunction of ETC in damaged mitochondrial leads to excessive ROS production, which is quite detrimental to cells, resulting in dopaminergic neuron death. ROS also triggers the autophagy/mitophagy process, with the consequent removal of damaged mitochondria, and in turn enhances cellular survival (Elfawy and Das, 2019). However, once the accumulation of ROS results from OS, proteins and toxic wastes can be deposited in the brain, thereby leading to dysfunction of the brain. Along with increased production of ROS, decreased production of antioxidant enzymes can together lead to neurodegeneration in PD. ROS can damage mtDNA by inducing mutations, leading to more dysfunction of OXPH and mitochondrial morphology, resulting in the vicious cycle of the mitochondria in PD (Elfawy and Das, 2019). Mitochondrial dysfunction also causes the decreased production of ATP, an influx of calcium, and the opening of the mitochondrial permeability pore, eventually resulting in apoptosis.

### NADPH Oxidases Activation and Oxidative Stress in PD

**FIGURE 4:**
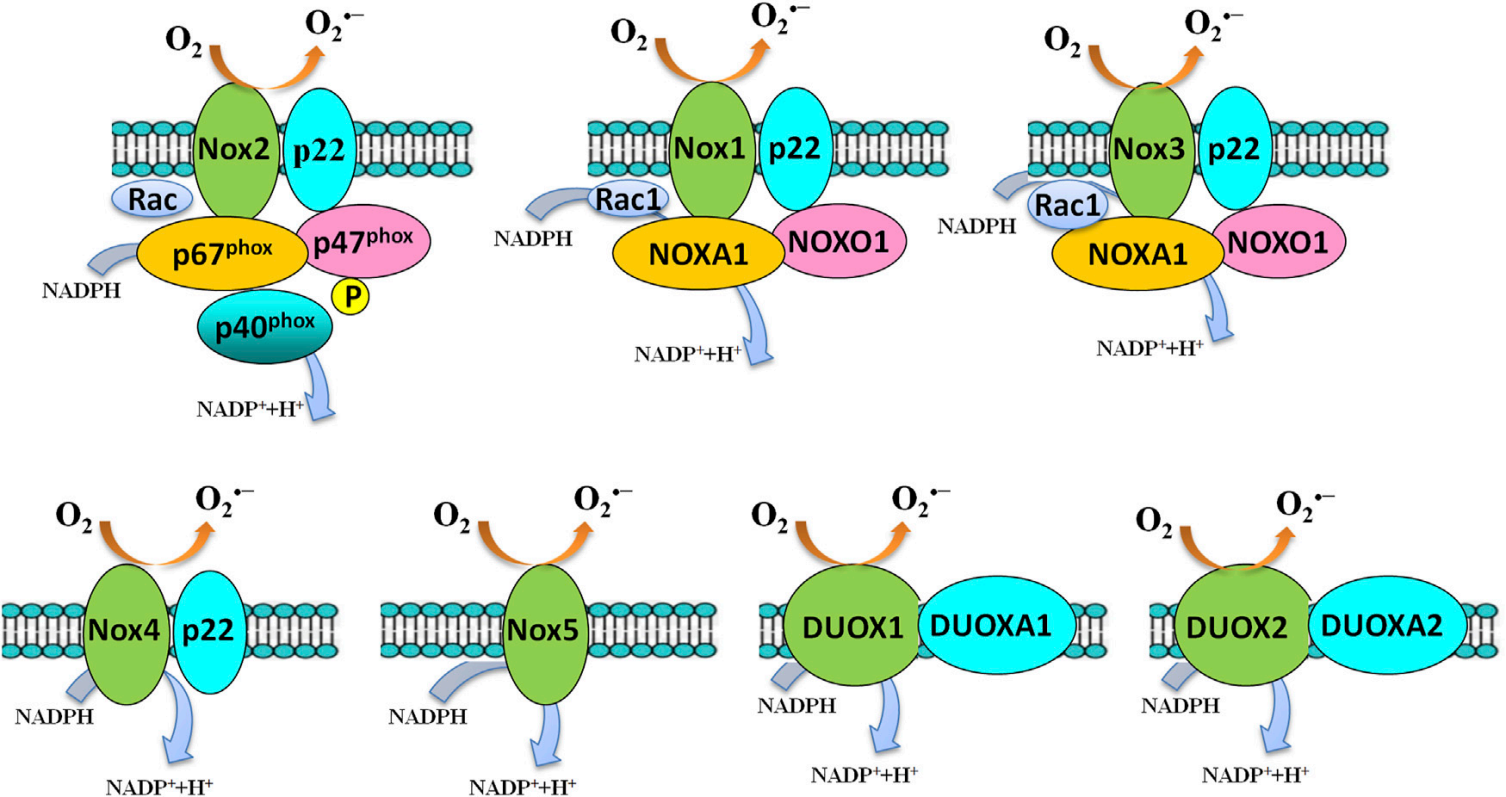
Activation of the NADPH oxidase family members. Several components and domains make up the transmembrane-active enzyme complexes of NADPH oxidase isoforms. NOX1-5 and DUOX1/DUOX2 are shown. Upon activation, an electron will be transferred from NADPH to O_2_ to form superoxide. NOX4-generated superoxide undergoes rapid conversion into hydrogen peroxide, which mediates many of its downstream effects. NOX5 and the DUOX enzymes are sensitive to cellular Ca^2+^ concentrations.

NADPH oxidases are a family of membrane-bound, multisubunit enzyme complexes. The primary function of NADPH oxidases is to transfer electrons across the plasma membrane from NADPH to molecular oxygen *via* their “Nox” catalytic subunit to generate O_2_^•−^ and subsequently ROS, including H_2_O_2_ and HO (Figure 4) (Tarafdar and Pula, 2018). NADPH oxidases consist of two membrane-bound components and three components in the cytosol, plus rac 1 or rac 2. The NADPH oxidase family of enzymes, consisting of seven members in mammalian species (NOX2, NOX1, NOX3, NOX4, NOX5, DUOX1, and DUOX2-containing NADPH oxidases), was a major source of ROS that is important in diverse cellular functions, including antimicrobial defense, inflammation, and redox signaling (Bedard and Krause, 2007). According to the new terminology, the catalytic subunit of NADPH oxidases includes NOX2 (gp91phox), and its six homologs (NOX1, NOX3, NOX4, NOX5, DUOX1, and DUOX2) are referred to as the NOX family. These seven isoforms, sharing not only conserved functions but also conserved structural properties, are transmembrane proteins and primarily distinguished by the presence of the distinct membrane-spanning catalytic “Nox” (Nox1-Nox5) or “Duox” subunit (Duox1-Duox 2), which mediate the electron transfer process from NADPH to molecular oxygen (Vermot et al., 2021). The catalytic NOX subunits have unique distribution patterns and are widely expressed in different tissues throughout the body. Many cells express several NOX isoforms; differences in subcellular distributions and activation mechanisms of different NOX isoforms might explain the nonredundancy in their functions [for a review see: references (Bedard and Krause, 2007; Ma et al., 2017)].

NOX2-containing NADPH oxidases are the best-characterized member of the NADPH oxidase family of enzymes (Figure 4). NOX2-containing NADPH oxidases were firstly identified and discovered in studying a process called “respiratory burst” in neutrophils (Berendes et al., 1957; Sbarra and Karnovsky, 1959; Rossi and Zatti, 1964; Babior et al., 1973, 1975; Segal et al., 1978; Segal and Jones, 1978). Two research groups cloned the gene coding for the catalytic subunit of the phagocyte NADPH oxidase, i.e., the gp91^phox^ in the late 1980s (Royer-Pokora et al., 1986; Teahan et al., 1987). gp91^phox^ is now called NOX2 in the novel NOX terminology.

NADPH oxidases are activity-dependent, which activation usually requires the translocation of cytosolic subunits to the membrane-bound subunits p22^phox^ and NOX isoforms. NOX2 (gp91^phox^) is the best-characterized member of the NOX family. Once stimulation, the cytosolic subunits of NADPH oxidases, i.e., p47phox, p67phox, p40phox, and the small Rho GTPase, Rac1, or Rac2, translocate to the membrane-bound p22phox/NOX2 heterodimer to assemble the active NADPH oxidases complexes, which catalyzes the reduction of O_2_ to generate O_2_^•−^ and subsequently H_2_O_2_ and HO^•^.

**FIGURE 5:**
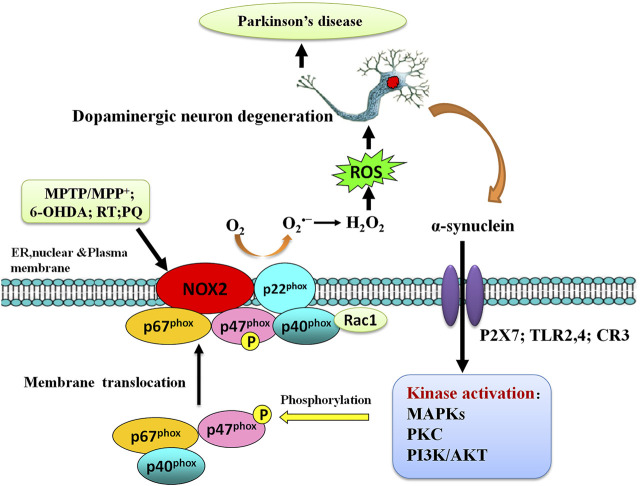
Alpha-synuclein and microglial Nox2 activation. The activation of microglia by alpha-synuclein can implicate several cell-surface receptors, such as P2X7, TLR2/4, and CR3, and subsequent activation of several kinases, such as PKC, Akt, MAPKs, PAK, and ERK1/2. This in turn could promote the phosphorylation and translocation of p47phox and subsequent Nox2 activation. Released oxygen species appear to promote microglia chemoattraction, activation, and OS. Neuronal damage leads to the release of alpha-synuclein and the TLR-agonist high mobility group box protein 1 (HMGB1).

Mounting evidence has shown that microglial NOX2 contributes to CNS OS and neuronal damage. NOX2-containing NADPH oxidases have emerged as a major source of OS in PD (Gao et al., 2003a; Qin et al., 2004; Wu et al., 2005; Kim et al., 2007; Gao et al., 2012; Marrali et al., 2018; Sun et al., 2020) (Figure 5). NOX2 is expressed in several regions of the brain and various cell types, including neurons at the striatum (McCann et al., 2008; Guemez-Gamboa et al., 2011), substantia nigra (Zawada et al., 2011; Qin et al., 2013), and midbrain (Qin et al., 2013), and is heavily expressed in microglia than in neurons and astroglia. Postmortem SN samples from brains of patients with PD had higher NOX2 protein content than samples from control individuals, and an increase of NOX2 was also observed in microglia in the ventral midbrain of MPTP-treated mice (Wu et al., 2003). The same study also showed that NOX2 is upregulated in SNpc of mice after repeated intraperitoneal injections of MPTP. The upregulation of NOX2 coincides with the local production of ROS, microglial activation, and DA neuronal loss. NOX2 knockdown abates MPTP-associated ROS production and shows less SNpc DA neuronal loss than their WT littermates (Wu et al., 2003). These findings support that microglial NOX2 is a common pathway for selective DA neurotoxicity. Since then, ample evidence has been well documented on the role of microglial NADPH oxidase activation in the pathogenesis of PD. This study was corroborated by numerous *in vitro* cell cultures lacking functional NOX2 failing to produce neurotoxicity induced by MPP^+^ (Kim et al., 2007; Zhang et al., 2008; Jiang T. et al., 2016), MPTP (Gao et al., 2003b, 2003c; Kim et al., 2007), paraquat (Wu et al., 2005), or rotenone (Gao et al., 2003a) and *in vivo* studies show that mice lacking NOX2 receiving MPTP (Kim et al., 2007), paraquat (Purisai et al., 2007), and 6-OHDA (Hernandes et al., 2013a; 2013b) are less sensitive to dopaminergic degeneration. Many studies have suggested that NADPH oxidase has been linked to microglia-derived OS after a variety of PD-related neurotoxin, for example, 6-OHDA (Rodriguez-Pallares et al., 2007), rotenone (Gao et al., 2002), paraquat (Wu et al., 2005), and α-synuclein (Zhang et al., 2005), which suggest that microglia are the major NOX2-expressing cells in PD and in PD experimental models. Microglial NADPH oxidase activation and NOX2-containing NADPH oxidases-derived ROS have been suggested to contribute to the injury to DA neurons in PD, which may be a common denominator associated with neurotoxicity in PD, and could contribute to its pathophysiology.

### Oxidative Stress Caused by Dopamine Autooxidation

**FIGURE 6:**
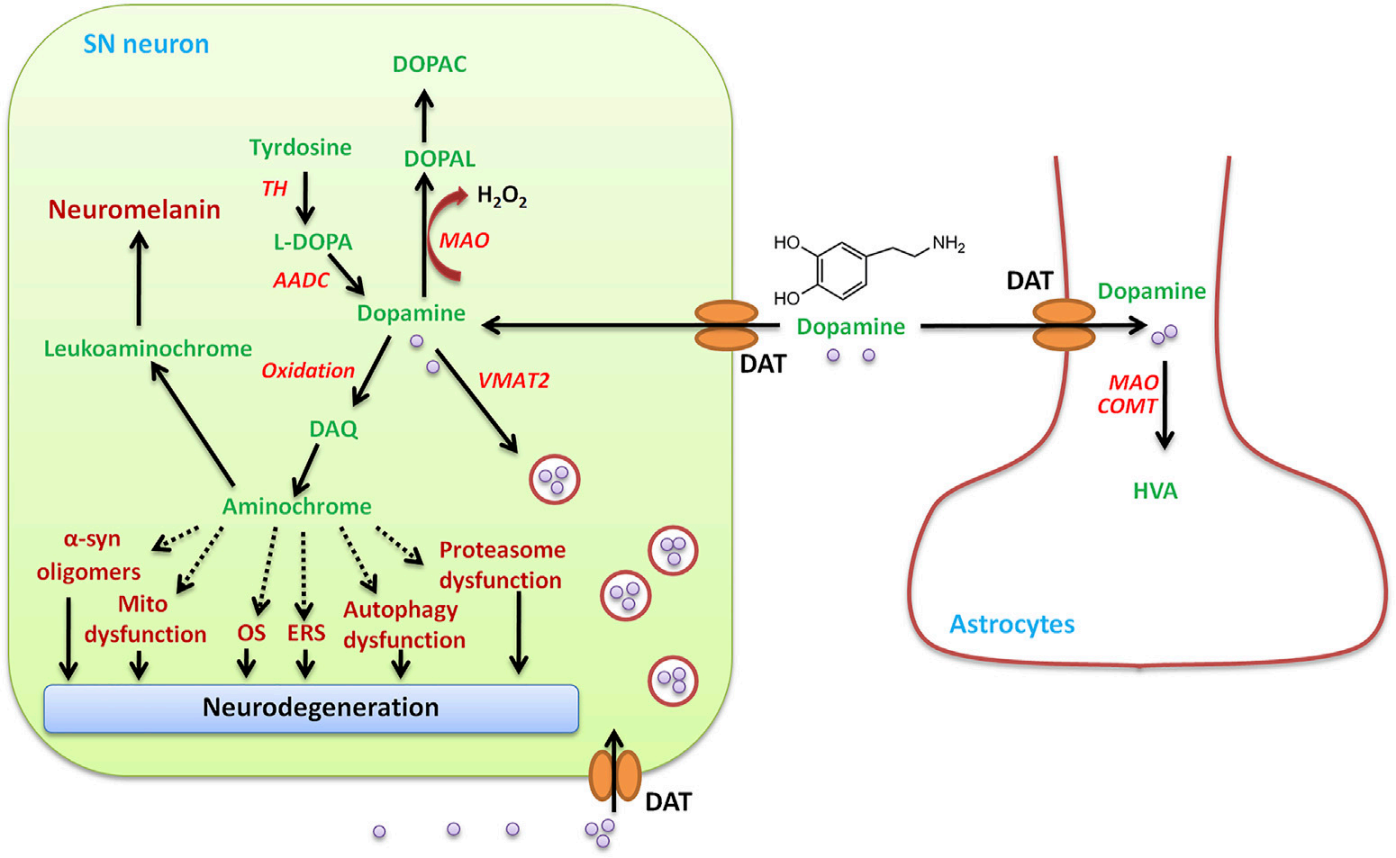
SN neuron DA metabolism. In addition to the uptake of DA by the DA transporter (DAT) from outside, DAergic neurons in the SN produce DA under the action of tyrosine hydroxylase (TH) and aromatic amino acid decarboxylase (AADC) in the cytosol. The amino acid tyrosine is converted to L-dopa using TH, and the second L-dopa is converted to dopamine using AADC. The newly synthesized or taken up DA is immediately transported to and stored in the monoaminergic vesicles by VMAT-2, preventing the existence of free dopamine in the cytosol. Cytosolic DA oxidizes to dopamine o-quinone (DAQ), which is immediately cyclized to aminochrome, which induces mitochondrial dysfunction, endoplasmic reticulum stress (ERS), oxidative stress (OS), the aggregation of alpha-synuclein, and the dysfunction of protein degradation. Dopamine in the cytosol can be degraded by monoamine oxidase-mediated degradation to 3,4-dihydroxyphenylacetaldehyde (DOPAL), hydrogen peroxide, and ammonia, which is converted to 3,4-dihydroxyphenylacetic acid (DOPAC) by aldehyde dehydrogenase. DA can be taken up into glial cells and degraded by catechol-o-methyltransferase (CMOT) or monoamine oxidase (MAO) to form homovanillic acid (HVA).

The pathological hallmarks of PD are selective degeneration of the DA neurons of the SN, which is more vulnerable ROS generated by the nigral DA neurons during dopamine metabolism (Chinta and Andersen, 2008), suggesting the possibility that dopamine itself may lead to the neurodegenerative process (Hastings, 2009). Under normal conditions, dopamine is synthesized from tyrosine in the cytosol, in which the phenol ring undergoes hydroxylation to levodopa under the catalysis of tyrosine hydroxylase (TH). The TH is the rate-limiting enzyme in dopamine biosynthesis (Figure 6). Levodopa is then decarboxylated to DA by the enzyme aromatic L-amino acid decarboxylase (AADC) (Napolitano et al., 2011; Segura-Aguilar et al., 2016). Once formed, dopamine is safely stored in high millimolar concentrations in synaptic vesicles after uptake by vesicular monoamine transporter 2 (VMAT2) (Miesenböck et al., 1998; Staal et al., 2004). TH and AADC are associated with VMAT-2 generating a complex.

Excess DA that is not stored in vesicles by VMAT2 will undergo either degradation or oxidation in the cytosol (Zhang B. et al., 2019). The MAO-mediated degradation of DA produces H_2_O_2_, which leads to OS in PD. DA eventually degrades to homovanillic acid (HVA) under the action of monoamine oxidase-B (MAO-B) and catechol-o-methyltransferase (COMT), producing H_2_O_2_ (Zhang S. et al., 2019). Once the excitation of DAergic neurons, dopamine in synaptic vesicles is released into the synaptic cleft from dopaminergic axon terminals and then binds to its receptors that are localized in postsynaptic dendrites/neurons (Werkman et al., 2006; Zhang B. et al., 2019). At a later stage, the excitation signal is terminated and the extracellular free DA is removed from the synaptic cleft by the dopamine transporter (DAT) expressed on the dopaminergic nerve endings and can be reutilized by DAergic neurons or taken up by astrocytes. The DA that DAT-mediated took up in DAergic neurons is sequestered by VMAT2 into synaptic vesicles. DA leaking from synaptic vesicles accumulates in the cytosol and then is degraded by MAO-B, producing hydrogen peroxide and 3,4-dihydroxyphenylacetaldehyde (DOPAL) (Zucca et al., 2017), which is then reduced to inactive 3,4-dihydroxyphenylethanol (DOPET) or further oxidized to 3,4-dihydroxyphenylacetic acid (DOPAC) by alcohol dehydrogenase (ADH) or acetaldehyde dehydrogenase (ALDH) (Herrera et al., 2017). Astrocytes can also take up DA in the synaptic cleft and easily degrade DA by MAO and COMT, which catalyzes the methylation of DOPAC to finally form HVA, the main product of DA degradation (Inyushin et al., 2012).

DA autooxidation, another source of OS to DAergic neurons, forms ROS and reactive o-quinones, which include DA-o-quinone (DAQ) and aminochrome (Zucca et al., 2017). When free DA in the cytosol of DA neurons exceeds the physiological content, DA can oxidize to DAQ, where they finally polymerize, producing neuromelanin (Segura-Aguilar et al., 2014; Herrera et al., 2017), which immediately cyclizes to aminochrome (Herrera et al., 2017). Aminochrome then participates in neurotoxic reactions by inducing chronic neurotoxicity in the dopaminergic neurons. Aminochrome can result in α-synuclein modification (generating neurotoxic oligomers), mitochondrial dysfunction, OS, autophagy dysfunction, proteasomal dysfunction, and endoplasmic reticulum stress (Herrera et al., 2017), all of which are related to cellular changes in PD.

### Iron Accumulation (Fe^2+^) and Oxidative Stress in PD

Specifically increased content of iron in SN is another common hallmark of PD brains, suggesting the possibility that iron may contribute to the selective degeneration of the DA neurons in the SN. Lhermitte’s pioneering study has shown the occurrence of abnorma1 iron deposits in the brain of PD patients (Lhermitte et al., 1924). After that pioneering study, accumulating evidence suggests that iron accumulation results in OS in PD. A more detailed description of the molecular mechanism by which iron leads to OS in PD is seen in other reviews (Ke and Qian, 2007; Weinreb et al., 2013; Belaidi and Bush, 2016; Xu et al., 2017; Zucca et al., 2017; Chen et al., 2019).

## Nrf2/ARE Pathway and PD

### Concise Overview of Keap1/Nrf2/ARE Pathway

Based on previous works, targeting Keap1/Nrf2/ARE pathway is becoming a strong candidate for therapy for neurodegenerative disease (Zgorzynska et al., 2021). As a core factor, Nrf2 orchestrates the cytoprotective pathway and regulates the expression of several protective genes containing AREs in their promoters, which function to restore homeostasis after combatting OS (Bento-Pereira and Dinkova-Kostova, 2021). Nrf2 was discovered as a member of the human cap’n’collar (CNC) basic-region leucine zipper transcription factor family in 1994 (Moi et al., 1994). In the nucleus, NRF2 forms complexes with small musculoaponeurotic fibrosarcoma protein (MAF) K, G, and F, which recognizes and is bound to an enhancer sequence termed ARE; the latter is present in the regulatory regions of over 250 genes (i.e., ARE genes) (Cuadrado et al., 2018). In unstressed conditions, KEAP1 and CULLIN3 (CUL3) form a ubiquitin E3 ligase complex in the cytoplasm, which polyubiquitinates NRF2 for rapid degradation through the proteasome system (Yamamoto et al., 2018). NRF2 is synthesized but constantly degraded. KEAP1 was identified as a repressor of Nrf2 in 1999 (Itoh et al., 1999). KEAP1 functions were identified as a sensor, while NRF2 plays a role as an effector for the coordinated activation of cytoprotective genes in the KEAP1/NRF2 system. Nrf2 regulates the expression of a battery of cytoprotective genes involved in several cellular processes, such as xenobiotic metabolism and detoxification, ROS scavenging, glutathione, NADPH homeostasis, and autophagy (Bento-Pereira and Dinkova-Kostova, 2021).

The Keap1/Nrf2/ARE signaling pathway is primarily regulated by Keap1-dependent and Keap1-independent mechanisms [more detail seen in other reviews (Bryan et al., 2013; Zhang et al., 2013; Tebay et al., 2015; Zenkov et al., 2017)]. In brief, the activity of Nrf2 is primarily regulated by Keap1, through its interaction with Keap1 which directs the transcription factor for proteasomal degradation. OS or exposure to electrophilic agents can react with Keap1 and stabilize Nrf2, leading to nuclear accumulation of Nrf2 and upregulated Nrf2 protein levels. Once in the nucleus, Nrf2 dimerizes with small Maf proteins and binds to the ARE, leading to transcriptionally driving the expression of several protective genes. The alternative Keap1-independent regulation mechanisms of Nrf2 include protein kinases-induced phosphorylation of Nrf2, interaction with other protein partners, and epigenetic factors (Zhou et al., 2019). Human Nrf2 contains a large number of serine, threonine, and tyrosine residues (17%), which can be phosphorylated by the protein kinases, which belong to various families, including PKC, JNK, PI3K, ERK, p38 MAPK, PERK, AMPK, and GSK-3β, all of which participate in regulating Nrf2 stability and translocation into the nucleus and bind to ARE (Zenkov et al., 2017; Rai et al., 2019b).

Growing experimental evidence implicates that the Keap1/Nrf2 system serves as an attractive drug development target in PD. Nrf2 may play several significant roles in mitochondrial function, which provides a potential therapeutic target for mitochondrial dysfunction in PD. Activation of Nrf2 by natural bioactive compounds is a promising approach for PD.

### Role Played by Nrf2 During PD

The pioneering studies of Johnson and colleagues have provided the proof of concept that activation of Nrf2 protects cells and animal models against OS-associated neurodegeneration and revealed appropriate strategies for induction of Nrf2 through pharmacologic modulation to combat OS (Lee et al., 2003a; Shih et al., 2003; Kraft et al., 2004; Johnson et al., 2008; Calkins et al., 2009). Systematic Nrf2 deficiency sensitizes neurons to 3-NP toxicity in cell culture and in whole animals (Calkins et al., 2005). Nrf2 knockout mice are significantly more sensitive to mitochondrial complex I and II inhibitors (Johnson et al., 2008).

Recent evidence has proven that Nrf2 is a novel neuroprotective platform that rendered resistance to a variety of PD-related OS-dependent neurotoxin insults. Regarding PD, evidence from Nrf2 deficiency in cell and animal models supports the functional importance of Nrf2 during PD. Nrf2 protects mixed primary astrocytes and neurons through coordinate upregulation of ARE-driven genes. Nrf2^-/-^ neurons in primary neuronal cultures containing both astrocytes and neurons were more sensitive to MPTP or rotenone (Lee et al., 2003b). This observation was corroborated by further studies, which reported that Nrf2 deficiency exacerbates vulnerability to the 6-OHDA both *in vitro* and *in vivo (* Jakel et al., 2007). They further showed that tert-butylhydroquinone activates the Nrf2/ARE pathway and protects against 6-OHDA *in vitro*. Induction of Nrf2/ARE by transplantation of astrocytes overexpressed Nrf2 can protect living mice against 6-OHDA-induced damage (Jakel et al., 2007). Nrf2 deficiency increases the vulnerability to PD-related neurotoxin MPTP sensitivity *in vivo (* Chen et al., 2009). Using siRNA knockdown of Keap1, activation of the Nrf2/ARE pathway can reduce OS and partially provide protection against MPTP-mediated neurotoxicity (Williamson et al., 2012). Overexpression of Nrf2 in astrocyte delays synuclein aggregation and motor deficit throughout the CNS in the alpha-synuclein mutant (A53T) mouse model, suggesting that Nrf2 in astrocytes exerts neuroprotection from hSYN(A53T)-mediated toxicity through promoting the degradation of hSYN(A53T) *via* autophagy-lysosome pathway *in vivo*. Thus, activation of the astrocytes Nrf2 is a potential target to develop therapeutic strategies for treating PD (Gan et al., 2012). Collectively, these studies suggest that the Nrf2/ARE pathway is a promising target for therapeutics in PD (Jakel et al., 2007).

## Nrf2/ARE/HO-1 Pathway and Therapeutic Modulation of Parkinson’s Disease

### Neuroprotective Role of the Activation of Nrf2 in PD

Mounting evidence indicates that activators of the Nrf2/ARE pathway displayed significantly greater resistance to neurotoxicity induced by 6-OHDA (Table 2), MPP^+^ (Table 3), MPTP (Table 4), paraquat (Table 5), and rotenone-induced (Table 6) *in vitro* or *in vivo* model. The presence of activation of Nrf2 by pharmacologic compounds was shown to exert neuroprotection, or conversely, Nrf2 deficiency led to exacerbating neuron sensitivity to the neurotoxin. It is becoming evident from the published literature that activation of Nrf2 can protect against PD-related neurotoxin-induced neurotoxicity when activated before or coincident with neurotoxin exposure. Targeting Nrf2 activity is emerging as a strong candidate for the treatment of PD.

**TABLE 2:** Summary of the experimental studies involving compounds able to modulate the Nrf2 pathway in 6-OHDA-induced PD models.

References	Compound	Compound dose	Toxin	Toxin dose	Model	Nrf2	Signaling	ARE gene
Sheng et al. (2021)	SDA	20–30%	6-OHDA	60 µM	PC12	+	ND	↑HO-1(P)
Wu et al. (2021)	Fucoxanthin	1–5 µM	6-OHDA	250 µM	PC12	+	↓Nrf2-Keap1 binding	↑GCLC, GCLM, and HO-1(P)
Zhang et al. (2021)	Ginnalin A	10–20 µM	6-OHDA	100 µM	SH-SY5Y	+	ND	↑GCLC, HO-1, and NQO1(P, M)
Sano et al. (2021)	Fluprostenol	100–500 mM	6-OHDA	50 µM	SH-SY5Y	+	ERK	↑GCLM, HO-1, and NQO1(M)
Ma et al. (2020)	Isoorientin	5–20 µM	6-OHDA	300 µM	SH-SY5Y	+	AMPK; PI3K/AKT	↑GCLC, GCLM, HO-1, NQO1, and Trx-1(P)
Kaji et al. (2020)	Sesaminol	1 μg/ml	6-OHDA	20 µM	SH-SY5Y	+	ND	↑Activities of NQO1
Ji et al. (2021)	Piperlongumine	5–20 µM	6-OHDA	150 µM	PC12	+	ND	↑NQO1, HO-1, GCLC, GCLM, and TrxR1(P)
Chen et al. (2020)	T-006	3–30 µM	6-OHDA	30 µM	DA neuron	+	Akt/GSK3β	↑HO-1(P)
Darabi et al. (2019)	Trehalose	-	6-OHDA	25 μg	Wistar rats	+	ND	Activities of GR and GPX
Anis et al. (2020)	Perillyl alcohol	100 mg/kg BW	6-OHDA	-	Male Wistar rats	+	ND	ND
Colonnello et al. (2020)	Caffeic acid	100 µM	6-OHDA	100 µM	Rat cortical slices	+	ND	↑Nrf2/ARE binding activity
Colonnello et al. (2020)	Caffeic acid	25 mM	6-OHDA	25 mM	*C. elegans*	+	ND	↑Nrf2/ARE binding activity
Kwon et al. (2019)	Hyperoside	0.5–2 µM	6-OHDA	200 µM	SH-SY5Y	+	ND	↑HO-1(M, P)
Betharia et al. (2019)	ACDT	25–50 µM	6-OHDA	40 µM	SH-SY5Y	+	ND	↑NQO1(P)
Xu L. L. et al. (2019)	Andrographolide	5–12.5 µM	6-OHDA	900 µM	PC12	+	↓neuroinflammation	↑HO-1(P)
Ren et al. (2019)	Tricetin	20–80 µM	6-OHDA	200 µM	SH-SY5Y	+	ND	↑HO-1(P)
Feng et al. (2019)	Stellettin B	0.1 nM	6-OHDA	20 µM	SH-SY5Y	+	PI3K/Akt	↑HO-1(P)
Yan et al. (2019)	Selenepezil	5–10 µM	6-OHDA	200 µM	SH-SY5Y	+	ND	↑GCLC, GCLM, HO-1,NQO1, and TrxR(P)
Zhang B. et al. (2019)	Icariin	60 mg/kg BW	6-OHDA	4 µg	Mice	+	↓neuroinflammation	↑GCLC, NQO1, and HO-1
Zhu J. L. et al. (2019)	Icariin	0.005–0.05 µM	6-OHDA	100 µM	PC12	+	ND	↑GCLC, NQO1, and HO-1
Eo et al. (2019)	Ukgansan	0.1–10 μg/ml	6-OHDA	75 µM	PC12	+	ND	↑NQO1(P)
Darabi et al. (2018)	SMER28	50 μg/kg BW	6-OHDA	12.5 µg	Wistar rats	↑Activity	ND	↑Activities of GSH, GPX, and SOD
Li C. et al. (2018)	Acteoside	100–400 μg/ml	6-OHDA	250 µM	Zebrafish	+	ND	↑GCLC, GCLM, HO-1, and NQO1(M). ↑Activities of CAT, GPX, and SOD
Chandrasekhar et al. (2018)	Gallic acid	1 μg/ml	6-OHDA	50 µM	SH-SY5Y	+	ND	↑Activities of CAT, GPX, SOD, and GR
Wu J. et al. (2018)	Protodioscin	5–20 mg/kg BW	6-OHDA	8 µg/time × 8 weeks	Wistar rats	+	ND	ND
Morroni et al. (2018)	Sulforaphane	5 μM	6-OHDA	100 µM	SH-SY5Y	+	ND	↑Activities of GSH
Morroni et al. (2018)	Erucin	5 μM	6-OHDA	100 µM	SH-SY5Y	+	ND	↑Activities of GSH
Funakohi-Tago et al. (2018)	Hydroxytyrosol butyrate	5–10 µM	6-OHDA	100 µM	SH-SY5Y	+	ND	↑HO-1(P + M)
Hou et al. (2018)	Honokiol	5–10 µM	6-OHDA	200 µM	PC12	+	ND	↑HO-1(P), NQO1, Trx, and TrxR(P)
Lee et al. (2018)	Sesquiterpenoid	5–10 µM	6-OHDA	250 µM	PC12	+	ND	↑HO-1(P/M)
Moon et al. (2018)	Carbon monoxide	100 µM	6-OHDA	150 µM	C6 glioma cells	+	ND	↑HO-1(P/M); ↑SOD(P/M)
Izumi et al. (2018)	TPNA10168	10 µM	6-OHDA	250 µM	PC12	+	Akt	↑HO-1(P/M); ↑γ-GCS(P); ↑ NQO1(P)
Inoue et al. (2018)	HPO-DAEE	10 µM	6-OHDA	70 µM	SH-SY5Y	+	ND	↑HO-1(P/+M)
Kao et al. (2017)	1T3O	0.001∼1 µM	6-OHDA	20 µM	SH-SY5Y	+	Akt	↑HO-1(P/M)
Peng et al. (2017)	Cardamonin	1–10 μM	6-OHDA	200 µM	PC12	+	ND	↑HO-1, NQO1, Trx1, and Trx1R(P)
Masaki et al. (2017)	DDC	1 nM	6-OHDA	2 μg/μl	C57BL/6N mice	+	ND	↑HO-1(P)
Masaki et al. (2017)	DDC	3–30 μM	6-OHDA	50 µM	PMC	+	ND	↑HO-1 and NQO1(P + M)
Murakami et al. (2018)	FPP	3 mg/ml	6-OHDA	12.5–100 µM	PCARE	+	ND	↑HO-1, NQO1, and GSH(P)
Pasban-Aliabadi et al. (2017)	Orexin-A	500 pM	6-OHDA	150 µM	SH-SY5Y	+	PKC; PI3K	ND
Feng et al. (2016)	11-de	10 nM	6-OHDA	150 µM	SH-SY5Y	+	PI3K/Akt	↑HO-1 and SOD(P)
Baluchnejadmojarad et al. (2017)	Ellagic acid	50 mg/kg	6-OHDA	2.5 μg/μM	Wistar rats	+	ND	↑HO-1 (ELISA)
Kim et al. (2017)	Capillarisin	10–50 µM	6-OHDA	150 µM	SH-SY5Y	+	JNK	↑HO-1(P + M); ↑Prx(P); ↑Trx(P); ↑NQO1(P)
Jing et al. (2016)	Tanshinone I	2.5–5 µM	6-OHDA	100 µM	SH-SY5Y	+	ND	↑HO-1(P); ↑ GCLC(P); ↑GCLM(P)
Yang et al. (2015)	PACA	5–50 µM	6-OHDA	200 µM	PC12	+	ND	↑HO-1(P); ↑NQO1(P); ↑ GCLC(P); ↑GCLM(P)
Peng et al. (2015a)	PLA4	20 µM	6-OHDA	200 µM	PC12	+	ND	H↑O-1, Trx1, TrxR1, NQO1, GCLC, and GCLM(M)
Peng et al. (2015a)	PLA5	20 µM	6-OHDA	200 µM	PC12	+	ND	↑HO-1, Trx1, TrxR1, NQO1, GCLC, and GCLM(M)
Peng et al. (2015b)	Hydroxytyrosol	10–50 μM	6-OHDA	200 µM	PC12	+	ND	↑HO-1, NQO1, and Trx1R(P)
Ju et al. (2015)	Chondroitin sulfate	200–800 mg/L	6-OHDA	50 µM	SH-SY5Y	+	ND	↑HO-1. ↑Activities of CAT, GSH, and SOD
Park et al. (2014)	α-Iso-cubebene	20 µM	6-OHDA	200 µM	SH-SY5Y	+	PKA; PKB	↑HO-1 and NQO1(P)
Lou et al. (2014)	Naringenin	20–80 μM	6-OHDA	200 µM	SH-SY5Y	+	ND	↑HO-1, GCLC, and GCLM(P)
Jing et al. (2015)	Dimethyl fumarate	1–4 μM	6-OHDA	100 µM	SH-SY5Y	+	ND	↑HO-1, NQO1, GCLC, and GCLM(P + M)
Meng et al. (2013)	NGR2	10–40 µM	6-OHDA	50 µM	SH-SY5Y	+	MEK1/2; ERK1/2	↑Activities of HO-1, GPX, and GR
Ryu et al. (2013)	Phloroglucinol	5–20 μg/ml	6-OHDA	90 µM	SH-SY5Y	+	Akt	↑CAT and GPX(P)
Deng et al. (2012a)	Sulforaphane	1–5 µM	6-OHDA	80 µM	PC12	+	ND	ND
Siebert et al. (2009)	tBHQ	5 µM	6-OHDA	100 nM	ONC	+	ND	↑NQO1(M)
Siebert et al. (2009)	Sulforaphane	5 µM	6-OHDA	100 nM	ONC	+	ND	↑NQO1(M)
Fujita et al. (2008)	Alpha-lipoic acid	300 µM	6-OHDA	75 µM	PC12	+	ND	↑Activities of GSH
Jakel et al. (2007)	tBHQ	10 µM	6-OHDA	75 µM	N27 cells	+	ND	↑HO-1 and NQO1(M)
Yamamoto et al. (2007)	Lactacystin	0.2–1 µM	6-OHDA	150 µM	PC12	+	p38 MAPK	↑Activities of GSH; ↑γ-GCS(M)
Zhang X. S. et al. (2015)	Tanshinone IIA	5–80 μg/ml	6-OHDA	100 µM	SH-SY5Y	+	ND	↑HO-1(P/M)
Zhang X. S. et al. (2015)	PCA	0.5–1 µM	6-OHDA	100 µM	PC12	+	ND	↑HO-1(P/M)
Zhang Z. et al. (2015)	Chrysin	12 µM	6-OHDA	100 µM	PC12	+	ND	↑HO-1(P/M)
Jin et al. (2015)	Pinocembrin	5–25 µM	6-OHDA	50 µM	SH-SY5Y	+	ND	↑HO-1(P); ↑γ-GCS(P)
Zhang et al. (2014)	Urate	200–400 µM	6-OHDA	50 µM	SH-SY5Y	+	ND	↑HO-1(P/M); ↑ GCLC(P)
Wang L. et al. (2014)	Carvedilol	10–20 µM	6-OHDA	100 µM	PC12	+	Akt	↑HO-1(P/M); ↑ NQO1(P)
Gunjima et al. (2014)	DBL	10–20 µM	6-OHDA	30 µM	SH-SY5Y	+	PI3K/Akt	↑HO-1(P/M); ↑ NQO1(P)
Chong et al. (2013)	Danshensu	200–400 µM	6-OHDA	250 µM	PC12	+	PI3K/Akt	↑HO-1(P)
Bae et al. (2013)	Berberine	10 µM	6-OHDA	60 µM	SH-SY5Y	+	PI3K/Akt; p38	↑HO-1(P)
Zhang C. t al. (2017)	Berberine	0.25–2 µM	6-OHDA	250 µM	PC12	+	PI3K/Akt	↑HO-1(P)
Lin et al. (2012)	Desipramine	10–20 µM	6-OHDA	50 µM	MDC	+	ERK; JNK	↑HO-1(P/M)
Oh et al. (2013)	SRE	10–50 µM	6-OHDA	200 µM	SH-SY5Y	+	ND	↑HO-1(P)
Izumi et al. (2012)	DDC	3–30 µM	6-OHDA	200 µM	PC12	+	PI3K/Akt; p38	↑HO-1(P)
Kim S.S. et al. (2012)	IGF-1	1–100 nM	6-OHDA	25 µM	PC12	+	ND	↑HO-1(P)
Kim Y. et al. (2012)	Licochalcone E	5 µM	6-OHDA	100 µM	SH-SY5Y	+	ND	↑HO-1(P/M); ↑ NQO1(P/M)
Deng et al. (2012b)	Sulforaphane	5 µM	6-OHDA	80 µM	PC12	+	PI3K/Akt	↑HO-1(P)
Hara et al. (2011)	Thapsigargin	0.3–30 µM	6-OHDA	80 µM	SH-SY5Y	+	ND	↑HO-1(M)
Hwang and Jeong (2010)	Ginsenoside Rb1	30–100 μg/ml	6-OHDA	50 µM	SH-SY5Y	+	PI3K/Akt	↑HO-1(P/M)
Hwang and Jeong (2008)	Kahweol	5–10 μM	6-OHDA	50 µM	SH-SY5Y	+	PI3K; p38	↑HO-1(P/M)
Hwang et al. (2008)	Metallothionein-III	25–50 ng/ml	6-OHDA	50 µM	SH-SY5Y	+	PI3K; ERK	↑HO-1(P/M)
Li et al. (2007)	tBHQ	40 μM	6-OHDA	100 µM	PC12	+	ND	↑HO-1(P/M)
Hara et al. (2006)	Apomorphine	20–30 µM	6-OHDA	50 µM	SH-SY5Y	+	ND	↑HO-1(M)
Zhang et al. (2012)	Baicalein	50–200 μM	6-OHDA	100 µM	PC12	+	PKCα; PI3K/AKT	↑HO-1(P/M)
Kurauchi et al. (2012)	CAPE	10–30 mg/kg	6-OHDA	2 μg/μM	Mouse	+	p38 MAPK	↑HO-1(P)
Hu et al. (2014)	Luteolin	20 μM	6-OHDA	100 µM	PC12	+	ND	↑HO-1(M); ↑ GCLC(M)
Kim et al. (2015)	DHC	0.4–10 μM	6-OHDA	100 µM	SH-SY5Y	+	ND	↑HO-1(P); ↑NQO1(P); ↑ GCLC(P)
Luo et al. (2017)	L-F001	1–10 μM	6-OHDA	200 µM	PC12	+	Akt/GSK-3beta	↑HO-1(P)
Ba et al. (2015)	Schisandrin B	100 μM	6-OHDA	100 µM	SH-SY5Y	+	ND	↑HO-1(P); ↑NQO1(P)

ND, not described; ACDT, disubstituted dithiolethione 5-amino-3-thioxo-3H-(1,2) dithiole-4-carboxylic acid ethyl ester; T-006, tetramethylpyrazine derivative; SDA, Shende’an tablet; HPO-DAEE, 4-hydroperoxy-2-decenoic acid ethyl ester; 1T3O, 1-tosylpentan-3-one; DDC, 2′,3′-dihydroxy-4′,6′-dimethoxychalcone; DFC, deferricoprogen; DMA. PACA, dimerumic acid, N-propargyl caffeate amide; PCA, protocatechuic acid; DBL, 3,4-dihydroxybenzalacetone; MDC, Mes23.5 dopaminergic cells; SRE, Sanguisorbae Radix extract; IGF-1, insulin-like growth factor-1; PCN, primary cortical neuron cultures; MGF24, 24-amino acid C-terminal peptide of mechano growth factor; lactacystin, a proteasome inhibitor; PMC, primary mesencephalic cultures; PSI, benzyloxycarbonyl-Ile-Glu(O-t-butyl)-Ala-leucinal; MG-132, benzyloxycarbonyl-Leu-Leu-leucinal; tBHQ, tert-butylhydroquinone; GLNVA, glyceryl nonivamide; NGF, Nerve Growth Factor; CAPE, caffeic acid phenethyl ester; SHXT, San-Huang-Xie-Xin-Tang; BNC, B35 neuroblastoma cells; DFE, *Drynaria fortunei* extract; DHC, 5,7-dihydroxychromone; NQO1, NAD(P)H:quinone1; Trx, thioredoxin; TrxR, thioredoxin reductase; SOD, superoxide dismutase; GCLC, glutathione cysteine ligase regulatory subunit; GLCM, glutathione cysteine ligase modulatory subunit; γ-GCS, γ-glutamylcysteine synthetase; Prx, peroxiredoxin; SMER28, 6-bromo-N-prop-2-enylquinazolin-4-amine, which is an autophagy inducer; GSH, glutathione; GPX, glutathione peroxidase; SCAE, sugarcane aqueous extract; CAT, catalase; GR, glutathione reductase; DDC, 2′,3′-dihydroxy-4′,6′-dimethoxychalcone from green perilla; PMC, primary mesencephalic cultures; FPP, fermented papaya preparation; PCARE, primary cultured astrocytes from rat embryos; 11-de,11-dehydrosinulariolide; NGR2, notoginsenoside R2; ONC, Organotypic Nigrostriatal Cocultures; PLA4, piperlongumine analogues 4; PLA5, piperlongumine analogues 5; n-3 PUFAs, omega-3 polyunsaturated fatty acids; P, protein; M, mRNA.

**TABLE 3:** Summary of the experimental studies involving compounds able to modulate Nrf2 pathway in MPP^+^-induced PD models.

References	Compound	Compound dose	Toxin	Toxin dose	Model	Nrf2	Signaling	ARE gene
Liu et al. (2021)	α-Lipoic acid	1–20 mM	MPP^+^	1 mM	PC12	+	PI3K/Akt	ND
Guo et al. (2021)	Irigenin	5–20 µM	MPP^+^	300 µM	BV-2 cells	+	ND	↑Activities of SOD, CAT, and GPx
Wang L. et al. (2020)	Ghrelin	1–10 µM	MPP^+^	1 mM	SH-SY5Y	+	ERK1/2	↑HO-1(P)
Li et al. (2020a)	Puerarin	3–10 µM	MPP^+^	250 µM	PC12	+	GSK-3β; Fyn	↑GCLC(P)
Zheng et al. (2020)	PGK1 inhibitor CBR-470-1	10 µM	MPP^+^	3 mM	SH-SY5Y	+	ND	↑HO-1, SOD1, and NQO1(P + M)
Li et al. (2020b)	Ferulic acid	3–10 µM	MPP^+^	250 µM	SH-SY5Y	+	ERK1/2	↑HO-1, GCLC, Trx1, and NQO1(P + M)
Yang et al. (2020)	Bruceine D	40–160 µM	MPP^+^	1 mM	MPCN	+	ND	↑GCLM and NQO1(P)
Wei et al. (2019)	NC001-8	100 nM	MPP^+^	1 mM	SH-SY5Y	+	ND	NQO1(P)
Guo C. et al. (2019)	Protocatechuic aldehyde	5–20 µM	MPP^+^	1 mM	SH-SY5Y	+	PLK2; p-GSK3β	ND
Zhu L. et al. (2019)	SC79	10 µM	MPP^+^	3 mM	SH-SY5Y	+	Akt	↑HO-1 and NQO1(P + M)
Li et al. (2019)	Salidroside	10–50 µM	MPP^+^	200 µM	MN9D cells	+	ND	↑SOD, GPx, and CAT(P)
Bao et al. (2019)	Sulforaphane	1–10 µM	MPP^+^	500 µM	PC12	+	ND	↑HO-1 and NQO1(P)
Guo X. et al. (2019)	Hydralazine	10 µM	MPP^+^	1 mM	SH-SY5Y	+	ND	↑HO-1, GCLC, GCLM, and NQO1(P)
Yan et al. (2018)	Simvastatin	1–1.5 µM	MPP^+^	100 µM	SH-SY5Y	+	ERK1/2	↑HO-1(P)
Chidambaram et al. (2018)	Cocoa beans	3–10 μg/ml	MPP^+^	2 µM	SH-SY5Y	+	ND	ND
Wu et al. (2017)	Salidroside	25–100 µM	MPP^+^	500 µM	SH-SY5Y	+	ND	↑SOD; GCLC(P + M)
Wang et al. (2017)	Thiazolidinedione	0.1–10 µM	MPP^+^	1 mM	SH-SY5Y	+	ND	ND
Lee et al. (2017)	2,4-Dinitrophenol	10 µM	MPP^+^	500 µM	PCNC	+	ND	ND
Zou Y. M. et al. (2015)	β-Ecdysterone	1–10 µM	MPP^+^	500 µM	PC12	+	Akt	↑HO-1(P + M)
Son et al. (2015)	KMS04014	1–10 µM	MPP^+^	1 mM	CATH.a cells	+	ND	↑NQO1(P + M)
Zhou et al. (2014)	Salvianolic acid B	10–100 µM	MPP^+^	1 mM	MCC	+	ND	ND
Alarcón-Aguilar et al. (2014)	tBHQ	10–50 µM	MPP^+^	25 µM	Cortical astrocytes	+	ND	↑γ-GCS(P); ↑GSH
Xiao et al. (2011)	Deprenyl	20–100 µM	MPP^+^	500 µM	PC12	+	PI3K/Akt; Erk	↑NQO1(P + M)
Li M. et al. (2018)	Pinostrobin	1–25 µM	MPP^+^	150 µM	SH-SY5Y	+	PI3K/AKT; ERK	↑HO-1(P)
Li X. et al. (2018)	FG-4592	50 µM	MPP^+^	350 µM	SH-SY5Y	+	ND	↑HO-1(P)
Jiang et al. (2014)	Gastrodin	1–25 µM	MPP^+^	100 µM	SH-SY5Y	+	P38MAPK	↑HO-1(P + M)
Jo et al. (2018)	Gintonin	50–100 mg/kg	MPP^+^	250 µM	SH-SY5Y	+	ND	↑HO-1(P)
Liu et al. (2017)	MT-20R	10–100 µM	MPP^+^	150 µM	CGNs	+	AKT	↑HO-1(P)
Ye et al. (2012)	Astaxanthin	5–20 µM	MPP^+^	500 µM	PC12	+	ND	↑HO-1(P)
Chen et al. (2012)	β-PGG	20–100 µM	MPP^+^	500 µM	PC12	+	AKT; ERK	↑HO-1(P + M)
Moreira et al. (2017)	TUDCA	100 µM	MPP^+^	100 µM	SH-SY5Y	+	ND	↑HO-1(P)
Wang et al. (2016)	Pinocembrin	10–30 µM	MPP^+^	200 µM	SH-SY5Y	+	ERK	↑HO-1(P + M)
Huang and Chuang (2010), Chuang et al. (2015)	FGF-9	10–100 ng/ml	MPP^+^	100 µM	PCN	+	AKT; ERK	↑HO-1(P + M)
Wruck et al. (2007)	Luteolin	20 μM	MPP^+^	100 µM	PC12	+	ERK	↑HO-1(M)

FG-4592, prolyl hydroxylase inhibitor; BCP, β-caryophyllene; PLE, *Paeonia lactiflora* extract; CGNs, cerebellar granule neurons; β-PGG, 1,2,3,4,6-penta-O-galloyl-β-D-glucose; TUDCA, tauroursodeoxycholic acid; NNCs, neocortical neuronal cells; FGF-9, fibroblast growth factor 9; PCNC, primary cortical neuron cultures; MGF24, 24-amino acid C-terminal peptide of mechano growth factor; MANF, mesencephalic astrocyte-derived neurotrophic factor; PLE, *Paeonia lactiflora* extract; DNC, dopaminergic neuron cultures; MPCN, mouse primary cortical neurons; MPP5, 3-methoxy-5-pentyl-phenol; MCC, mesencephalic cell culture; SC79, Akt activator; P, protein; M, mRNA.

**TABLE 4:** Summary of the experimental studies involving compounds able to modulate Nrf2 pathway in MPTP-induced PD models.

References	Compound	Compound dose	Toxin	Toxin dose	Model	Nrf2	Signaling	ARE gene
Zhao et al. (2021)	Withaferin A	20 μg/kg/day, i.p. × 7, 14 or 21 days	MPTP	25 mg/kg/day, i.p. × 7 days	C57BL/6 mice	+	ND	ND
Dong et al. (2021)	Paeoniflorin/glycyrrhetinic acid	50/50 mg/kg, p.o. × 2 weeks	MPTP	25 mg/kg/day, i.p. × 5 days	C57BL/6 mice	+	ERK1/2 and Akt	↑GCLM; GCLC(P)
Dutta et al. (2021)	Andrographolide	10 mg/kg/day, i.p. × 10 times	MPTP	20 mg/kg/day, i.p. on alternate days × 5 times	Male Swiss albino mice	+	p38 MAPK and ERK	ND
Sheng et al. (2021)	SDA	100–900 mg/kg, p.o. × 4 weeks	MPTP	30 mg/kg/day, i.p.) for 5 days	Male C57BL/6J mice	+	ND	↑HO-1 (P)
Dong et al. (2020)	Thymoquinone	10 mg/kg/day, i.p. × 7 d	MPTP	25 mg/kg/day, i.p. × 5 days	C57/BL6 mice	+	ND	↑HO-1, NQO1, and GST(P)
Huang et al. (2021)	PSP	30 mg/kg/day, p.o. × 4 weeks	MPTP	30 mg/kg/day, i.p. × 5 days	Male C57BL/6J mice	+	Akt	↑NQO1, HO-1, GCLM, and GCLC(P)
Choi et al. (2021)	Vinyl sulfones 9d	30 mg/kg/day, p.o. × 3 d	MPTP	20 mg/kg, i.p. four times at 2 h intervals	Male C57BL/6 mice	+	ND	↑NQO1, HO-1, GCLM, and GCLC(P + M)
Mohamed et al. (2021)	Tiron	140 and 280 mg/kg, i.p. × 10 days starting 5 days before MPTP injection	MPTP	30 mg/kg/day, i.p. × 5 days	Male albino mice	+	ND	↑HO-1(ICH)
Lin C. H. et al. (2020)	Trehalose	2% in drinking water	MPTP	30 mg/kg/day, i.p. × 15 times	Male C57BL/6 mice	+	ND	↑HO-1(P)
Li et al. (2020a)	Puerarin	15–60 mg/kg/day, p.o. × 14 d (3 d before MPTP)	MPTP	25 mg/kg/day, i.p. × 5 d	C57BL/6	+	GSK-3β; Fyn	↑GCLC(P)
Chen et al. (2020)	T-006	3–10 mg/kg/day, p.o. ×14 d	MPTP	30 mg/kg/day, i.p. × 5 d	Female C57BL/6 mice	+	Akt/GSK3β	↑HO-1(P)
Lee J. A. et al. (2020)	KKC080106	30 mg/kg, tid, p.o.	MPTP	20 mg/kg, i.p. four times at 2 h intervals	Male C57BL/6 mice	+	ND	↑NQO1, HO-1, GCLM, and GCLC(P + M)
Wang L. et al. (2020)	Piperine analogues-3b	50–100 mg/kg/day, p.o. × 7 d	MPTP	25 mg/kg/day, i.p. × 7 d	Male C57BL/6 mice	+	ND	↑HO-1; NQO1(P)
Li et al. (2020b)	Ferulic ACID	50 mg/kg/day, p.o. × 15 d	MPP^+^	25 mg/kg/day, i.p. ×5 d	C57BL/6J mice	+	ERK1/2	↑HO-1, GCLC, Trx1, and NQO1(P + M)
Yang et al. (2020)	Bruceine D	20–40 mg/kg/day, i.p. × 7 d	MPP^+^	15 mg/kg/day, i.p. × 7 d	MPCN	+	ND	↑GCLM; NQO1(P)
Zhao et al. (2020)	Rosmarinic acid	10–100 µM	MPTP	50 µM	Zebrafish embryos	+	ND	GCLM; NQO-1(P)
Kim et al. (2020)	KKPA4026	30 mg/kg/day, p.o. × 3 d	MPTP	20 mg/kg, i.p. four times at 2 h intervals	Male C57BL/6 mice	+	ND	↑GCLC, GCLM, NQO-1, and HO-1(P)
Guo C. et al. (2019)	Protocatechuic aldehyde	20 mg/kg/day, i.p. × 5 d	MPTP	30 mg/kg/day, i.p. × 7 d	Male C57BL/6 mice	+	PLK2;GSK3β	ND
Yang et al. (2019)	Astragaloside IV	40 mg/kg, oral gavage as described above for 7 days	MPTP	18 mg/kg, four times at 2 h intervals	Male C57BL/6 mice	+	ND	ND
Choi et al. (2019)	Compound 3c	20 mg/kg, p.o., 3 days	MPTP	20 mg/kg, i.p.; four times at 2 h intervals	Mice	+	ND	↑HO-1; GCLM(P + M)
Li et al. (2019)	Salidroside	15 and 50 mg/kg/day, 7 days	MPTP	30 mg/kg/day, i.p. × 5 d	Male C57BL/6 mice	+	ND	↑SOD, GPx, and CAT(P)
Guo X. et al. (2019)	Hydralazine	51.7 mg/kg per day by oral gavage for 3 weeks	MPTP	30 mg/kg/day, i.p. × 7 d	Male C57/BL6 mice	+	ND	↑HO-1, GCLC, GCLM, and NQO1(P)
Park et al. (2019)	β-Lapachone	5 mg/kg/day, i.p. × 3 d	MPTP	20 mg/kg, 4 times a day; 2 h interval	Male C57BL/6 mice	+	AMPK	↑HO-1(P)
Choi et al. (2018)	Kyung-Ok-Ko	2 g/kg/day	MPTP	20 mg/kg/day, i.p.	Male C57BL/6 mice	+	ND	↑HO-1; NQO1(P)
Xu Y. et al. (2019)	DDO-7263	50–100 mg/kg/day, i.p. × 10 d	MPTP	20 mg/kg/day, i.p. × 7 d	Male C57BL/6 mice	+	ND	↑HO-1; NQO1(P)
Wang G. et al. (2018)	Pramipexole	0.07–0.15 cm^2^ (TP)	MPTP	30 mg/kg, i.p.	C57BL/6 mice	+	ND	↑HO-1(P)
Li M. et al. (2018)	Pinostrobin	0.2–125 µM	MPTP	360 μM	Zebrafish	+	PI3K/AKT; ERK	↑HO-1(P)
Li X. et al. (2018)	FG-4592	10 mg/kg/day, i.p.	MPTP	30 mg/kg/day, i.p.	C57BL/6 mice	+	ND	↑HO-1(P)
Begum M and Sen (2018)	SNC-80	10 mg/kg	MPTP	30 mg/kg/day, i.p.	Swiss albino mice	+	ND	↑HO-1(P)
Jo et al. (2018)	Gintonin	50–100 mg/kg	MPTP	30 mg/kg/day, i.p. × 5 d	C57BL/6N mice	+	ND	↑HO-1(P)
Kabel et al. (2018)	Linagliptin	3–10 mg/kg/day	MPTP	*	80 Balb/c mice	+(ELISA)	ND	↑(ELISA)
Huang et al. (2017)	Uric acid	25 mg/kg/day × 13 d	MPTP	25 mg/kg/day, i.p. × 7 d	C57BL/6 mice	+	ND	HO-1(M)
Son et al. (2017)	Exemestane	1–10 mg/kg	MPTP	20 mg/kg, i.p. × 4 times	C57BL/6J mice	+	ND	↑GCLC, GCLM, HO-1, and NQO1(P + M)
Wang et al. (2017)	Thiazolidinedione	10–40 mg/kg	MPTP	30 mg/kg/day, i.p. × 5 d	C57BL/6 mice	+	ERK	ND
Meng et al. (2017)	Matrine	4–16 mg/kg	MPTP	30 mg/kg/day, i.p. ×4 d	C57BL mice	+	ND	↑Activities of SOD and GSH
Lee et al. (2017)	2,4-Dinitrophenol	1–5 mg/kg × 13 d	MPTP	20 mg/kg/2 h, i.p. × 4 times	C57BL mice	+	ND	ND
Smirnova et al. (2016)	NDGA	100 mg/kg/day	MPTP	10 mg/kg/2 h × 4 times	C57Bl6 mice	+	ND	ND
Ahuja et al. (2016)	DMF	100 mg/kg/day	MPTP	10 mg/kg	C57BL/6 mice	+	S-Alkylation of Keap1	↑GCLC, GCLM, HO-1, GSR, and NQO1(P)
Ahuja et al. (2016)	MMF	100 mg/kg/day	MPTP	10 mg/kg	C57BL/6 mice	+	S-Alkylation of Keap1	↑GCLC, GCLM, HO-1, GSR, and NQO1(P)
Moreira et al. (2017)	TUDCA	50 mg/kg × 3 d	MPTP	40 mg/kg	C57BL/6 mice	+	ND	↑HO-1(P); GPX (P + M)
Liu et al. (2017)	MT-20R	60–180 mg/kg × 7 d	MPTP	30 mg/kg/day, i.p. × 5 d	C57BL/6 mice	+	AKT	↑HO-1(P)
Luo et al. (2017)	L-F001	35–70 mg/kg × 7 d	MPTP	40 mg/kg	C57BL/6 mice	+	Akt/GSK-3beta	↑HO-1(P)
Ozkan et al. (2016)	DHA	36 mg/kg/day	MPTP	20 mg/kg	C57BL/6 mice	+	ND	↑HO-1(P)
Zhao J. et al. (2015)	Fasudil	20 mg/kg, bid × 7 d	MPTP	%	C57BL/6 mice	+	ND	↑HO-1(P)
Son et al. (2015)	KMS04014	30 mg/kg, qd × 3 d	MPTP	20 mg/kg, i.p. × 4 times	C57Bl/6 mice	+	ND	↑NQO1(P + M)
Zhao Y. F. et al. (2015)	Puerarin	50–150 mg/kg/day	MPTP	25 mg/kg/day, i.p. × 7 d	C57BL/6 mice	+	ERK1/2; PI3K/Akt	HO-1(P)
Wang L. et al. (2014)	Gastrodin	60 mg/kg/day, i.p. × 14 d	MPTP	30 mg/kg, qd, i.p. × 3 d	C57BL/6 mice	+	ERK1/2	↑HO-1; SOD (P + M)
Lee G. et al. (2016)	ITC-57	30 mg/kg × 3 d	MPTP	20 mg/kg/2 h, i.p. × 4	C57BL/6J mice	+	ND	HO-1(P + M)
Lee H. J et al. (2015)	VSC2	10 mg/kg/day × 3 d	MPTP	20 mg/kg, i.p. × 4	C57BL/6 mice	+	ND	HO-1(P + M)
Woo et al. (2014)	Vinyl sulfones	10 mg/kg	MPTP	20 mg/kg/2 h, i.p. × 4	C57BL/6 mice	+	ND	HO-1(P + M)
García et al. (2014)	S-Allyl cysteine	120 mg/kg, i.p. × 5 d	MPTP	30 mg/kg/day, i.p. × 5 d	C57BL/6 mice	+	ND	HO-1(P)
Zhou et al. (2014)	SalB	25 mg/kg, i.p. × 5 d	MPTP	20 mg/kg/2 h, i.p. × 4	C57BL/6J mice	+	ND	ND
Swanson et al. (2013)	Tetramethylpyrazine	20 mg/kg, i.p. × 7 d	MPTP	0.5 μm/μM	Wistar rats	+	ND	↑GCLC (P)
García et al. (2014)	S-Allyl cysteine	120 mg/kg, i.p. × 5 d	MPTP	30 mg/kg, i.p. × 5 d	C57BL/6J mice	+	ND	↑Activities of HO-1 and SOD
Galuppo et al. (2013)	RS-GRA	10 mg/kg	MPTP	40 mg/kg × 2	C57BL/6 mice	+	ND	ND
Kaidery et al. (2013)	Triterpenoids	4 μM	MPTP		C57Bl6 mice	+	ND	↑GCLC, GCLM, HO-1, and NQO1(P + M)
Yang et al. (2009)	CDDO-MA	50 mg/kg	MPTP	10 mg/kg/2 h, i.p. × 4	C57BL/6 mice	+	ND	↑GR, HO-1, and NQO1(P)
Jazwa et al. (2011)	Sulforaphane	50 mg/kg	MPTP	30 mg/kg	mice	+	ND	GCLC, HO-1, and NQO1(P + M)
Minelli et al. (2012)	Gly-Pro-Glu tripeptide	100 mg/kg	MPTP	4 mg/kg, i.p. × 4	C57BL/6 mice	+	ND	HO-1(P + M)

*MPTP: 8 mg/kg/day during the 1st week, 16 mg/kg/day during the 2nd week, 24 mg/kg/day during the 3rd week, and 32 mg/kg/day during the 4th week. %15 mg/kg bodyweight MPTP (Sigma, United States) dissolved in 0.9% saline on the 1st day, 20 mg/kg MPTP on the 2nd day, and 30 mg/kg MPTP daily next 5 days.

TP, transdermal patch; SNC-80, DOR agonist; L-F001, a multifunction ROCK inhibitor; DHA, docosahexaenoic acid; ITC-57, novel synthetic isothiocyanate; VSC2, (E)-1-(2-((2-methoxyphenyl)sulfonyl)vinyl)-2-chlorobenzene); PLGA, poly(lactic-co-glycolic) acid; DHB, the prolyl hydroxylase inhibitor 3,4-dihydroxybenzoate; HIF, hypoxia-inducible factor; NDGA, nordihydroguaiaretic acid; DMF, dimethylfumarate; MMF, monomethylfumarate; Gsr, glutathione reductase; SalB, salvianolic acid B; RS-GRA, (RS)-glucoraphanin, bioactivated with myrosinase enzyme; GR, glutathione reductase; CDDO-MA, 2-cyano-N-methyl-3,12-dioxooleana-1,9(11)-dien-28 amide; DDO-7263, 5-(3,4-difluorophenyl)-3-(6-methylpyridin-3-yl)-1,2,4-oxadiazole; PSP, *Polygonatum sibiricum* Polysaccharides; P, protein; M, mRNA.

**TABLE 5:** Summary of the experimental studies involving compounds able to modulate Nrf2 pathway in paraquat-induced PD models.

References	Compound	Compound dose	Toxin	Toxin dose	Model	Nrf2	Signaling	ARE gene
Rasheed et al. (2020)	Resveratrol		Paraquat			+	ND	↑ HO-1, NQO1, and Trx1(P)
Dos Santos Nunes et al. (2019)	Caffeic acid	0.25, 0.5, 1, and 2 mg/g of died × 7 days	Paraquat	0.44 mg/g of diet	*Drosophila melanogaster*	+	ND	ND
Srivastav et al. (2018)	BME	0.1–0.25%	Paraquat	20 mM	Drosophila	+	ND	ND
de Oliveira et al. (2017a); (2018a)	Carnosic acid	1 μM	Paraquat	100 μM	SH-SY5Y	+	ND	↑ HO-1(P)
Li et al. (2012)	tBHQ	Oral feeding	Paraquat	7 mg/kg	C57BL/6 mice	+	ND	↑ HO-1(P)
Li et al. (2012)	tBHQ	40 μM	Paraquat	100–300 μM	PC12	+	ND	↑ HO-1(P)
de Oliveira et al. (2017b)	Pinocembrin	25 μM	Paraquat	100 μM	SH-SY5Y	+	ERK1/2	↑ HO-1, GCLC, and GCLM(P)
Kobatake et al. (2017)	LG2055	1–100 μg/ml	Paraquat	0.5 mM	NIH-3T3 cells	+	JNK	↑HO-1, GCLC, GCLM, SOD, NQO1, and Txn1
de Oliveira et al. (2017c)	Tanshinone I	2.5 μM	Paraquat	100 μM	SH-SY5Y	+	ND	↑GPx, SOD, and γ-GCL(P)
de Oliveira et al. (2016)	Carnosic acid	0.1–0.5 μM	Paraquat	100 μM × 24 h	SH-SY5Y	+	PI3K/Akt	↑HO-1, GCLC, GCLM,SOD, NQO1, GR, and GPX
Lee J. A. et al. (2015)	DHA	25 μM	Paraquat	400 μM × 24 h	SN4741 cells	+	ND	↑GCLM and GR(M). ↑Activities of GSH
Minelli et al. (2009)	Cyclo (His-Pro)	50 μM	Paraquat	100 μM	PC12	+	p38 MAPK	↑HO-1, NQO1, GCLC, GCLM, GPX, GR, and Trx1(M)
Mizuno et al. (2011)	Sulforaphane	1 μM	Paraquat	200 μM × 24 h	Rat striatal cultures	+		↑ HO-1; γ-GCS
Mizuno et al. (2011)	6-HITC	1 μM	Paraquat	200 μM × 24 h	Rat striatal cultures	+		↑ HO-1; γ-GCS
de Oliveira et al. (2018b)	Naringenin	80 μM	Paraquat	100 μM	SH-SY5Y	+	ND	ND
Alural et al. (2015)	Lithium	2–5 mM	Paraquat	0.5 mM	SH-SY5Y	+	ND	HO-1(M)
de Rus Jacquet et al. (2017a)	Allium sativum	1–10 μg/ml	Paraquat	2.5 μM	PMC	+	ND	↑ HO-1(P + M)
de Rus Jacquet et al. (2017b)	Trifolium pratense	1–10 μg/ml	Paraquat	2.5 μM	PMC	+	ND	↑ HO-1(P + M)
de Rus Jacquet et al. (2017c)	Amelanchier arborea	1–10 μg/ml	Paraquat	2.5 μM	PMC	+	ND	↑ HO-1(P + M)

6-HITC, 6-(methysulfinyl)hexyl isothiocyanate, which is a naturally occurring isothiocyanate; tBHQ, tert-butylhydroquinone; PMC, primary midbrain cultures; Txn1, thioredoxin 1; BME, *Bacopa monnieri* extract; P, protein; M, mRNA.

**TABLE 6:** Summary of the experimental studies involving compounds able to modulate Nrf2 pathway in Rotenone-induced PD models.

References	Compound	Compound dose	Toxin	Toxin dose	Model	Nrf2	Signaling	ARE gene
Arab et al. (2021)	Dapagliflozin	1 mg/kg/day, po, every other day over 3 weeks	Rotenone	1.5 mg/kg, s.c., every other day over 3 weeks	Adult male Wistar rats		ND	↑Activities of HO-1
Thapa et al. (2021)	Suntamide A	1–10 μM	Rotenone	1 μM	SH-SY5Y	+	PI3K/AKT; ERK1/2	ND
Kaji et al. (2020)	Sesaminol	0.008%	Rotenone	10 mg/kg p.o. × 29 d	Male C57BL6/J mice	+	ND	↑Activities of NQO1
Wei et al. (2020)	Ellagic acid	100 mg/kg/days, p.o. × 35 d	Rotenone	1 mg/kg, s.c. 6 times a week for consecutive 5 weeks	C57BL/6J male mice	+	ND	↑HO-1 and NQO1(P)
El-Ghaiesh et al. (2020)	Metformin	100 or 200 mg/kg, every 24 ± 2 h, volume = 4 ml/kg	Rotenone	1 mg/kg, s.c. every 48 h, volume = 4 ml/kg	Male Swiss albino mice	+	ND	↑HO-1
Wang T. et al. (2020)	Danshensu	15–60 mg/kg, p.o. × 15 d	Rotenone	30 mg/kg	Male C57BL/6 mice	+	PI3K/AKT	↑HO-1, GCLC, and GCLM(P)
Garabadu and Agrawal (2020)	Naringin	80 mg/kg, i.p. × 14 d	Rotenone	2 μl into the right SNpc at a flow rate of 0.2 μl/min	Male Wistar albino rats	+	ND	↑Activities of Gr and GPx
Zhu L. et al. (2019)	SC79	10 µM	Rotenone	300 nM	SH-SY5Y	+	Akt	↑HO1 and NQO1(P + M)
Elmazoglu et al. (2020)	Luteolin	1–10 μM	Rotenone	20 μM × 12 h	BV2 cells	+	ND	↑Trx1(M)
Zhang et al. (2018)	Fucoidan	140 mg/kg/d × 38 d	Rotenone	1.5 mg/kg/d, 5 times/w × 5 w	SD rat	+	ND	ND
González-Burgos et al. (2017)	Ginsenosides Rd	0.5–50 μM	Rotenone	50 μM × 24 h	SH-SY5Y	+	ND	↑Activities of SOD
González-Burgos et al. (2017)	Ginsenosides Re	0.5–50 μM	Rotenone	50 μM × 24 h	SH-SY5Y	+	ND	↑Activities of SOD
Fernández-Moriano et al. (2017)	Ginsenosides Rb1	2.5–50 μM	Rotenone	50 μM × 24 h	SH-SY5Y	+	ND	↑Activities of SOD and GSH
Fernández-Moriano et al. (2017)	Ginsenosides Rg1	2.5–50 μM	Rotenone	50 μM × 24 h	SH-SY5Y	+	ND	↑Activities of SOD and GSH
de Rus Jacquet et al. (2017a)	Allium sativum	1–10 μg/ml	Rotenone	20 nM	PMC	+	ND	↑HO-1(P + M)
de Rus Jacquet et al. (2017b)	Trifolium pratense	1–10 μg/ml	Rotenone	20 nM	PMC	+	ND	↑HO-1(P + M)
de Rus Jacquet et al. (2017c)	Amelanchier arborea	1–10 μg/ml	Rotenone	20 nM	PMC	+	ND	↑HO-1(P + M)
Liu et al. (2016)	PF/β-Ecd	4–3.2 μM/0.4–3.2 μM	Rotenone	1 μM × 24 h	PC12	+	Akt	↑HO-1(P + M)
Michel et al. (2017)	TTMP	2 mg/kg, i.p. × 4 w	Rotenone	2 mg/kg, s.c. × 4 w	SD rat	+	ND	↑HO-1(P)
Gaballah et al. (2016)	Resveratrol	20 mg/kg/d, p.o. × 3 w	Rotenone	1.5 mg/kg, s.c. × 3 w	Wistar albino rats	+	ND	↑Activities of GPX
Cui et al. (2016)	Curcumin	100 mg/kg, bid, i.g. × 50 d	Rotenone	1 ml/kg/d, bid, i.g. × 50 d	Lewis rats	+	Akt	↑HO-1; NQO1(P). ↑Activities of GSH
Minelli et al. (2009)	Cyclo (His-Pro)	50 μM	Rotenone	100 μM	PC12	+	p38 MAPK	↑HO-1, NQO1, GCLC, GCLM, GPX, GR, and Trx1(M)
Zakharova et al. (2018)	rhLF	25 mg/kg	Rotenone	2.75 mg/kg	Wistar rats	+	ND	↑HO-1(M)
Engel et al. (2018)	Duloxetine	2–5 μM	Rotenone	10 μM	SH-SY5Y	+	PI3K/Akt	↑HO-1(M)
Zhang C. et al. (2017)	20C	1–10 μM	Rotenone	4 μM	SH-SY5Y	+	PI3K/Akt	↑HO-1(P)
Zhang X. L. et al. (2017)	20C	1–10 μM	Rotenone	4 μM	PC12	+	PI3K/Akt; GSK3β	↑HO-1(P)
Pan et al. (2016)	Safranal	10–50 μg/ml	Rotenone	100 nM	PDC	+	ND	↑HO-1(P + M)
Zhou et al. (2016)	Sulforaphane	50 mg/kg	Rotenone	30 mg/kg	C57BL/6 mice	+	ND	↑HO-1(P)
Huang et al. (2016)	20C	0.01–1 μM	Rotenone	4 μM	PC12	+	ND	↑HO-1(P + M)
Cui et al. (2016)	Curcumin	100 mg/kg, bid × 50 d	Rotenone	1 mg/kg/d, bid × 46 d	Lewis rats	+	Akt	↑HO-1(P)
Jo et al. (2018)	Gintonin	50–100 mg/kg	Rotenone	200–500 nM	SH-SY5Y	+	ND	↑ HO-1(P)
Lin et al. (2012)	Desipramine	10–20 μM	Rotenone	3 μM	MDC	+	ERK; JNK	↑HO-1(P + M)
Dal-Cim et al. (2012)	Guanosine	1 mM	Rotenone/Oligo A	30 μM/10 μM	SH-SY5Y	ND	PI3K/Akt; GSK-3β	↑HO-1(P)
Parada et al. (2010)	PNU282987	1–10 μM	Rotenone/Oligo A	30 μM/10 μM	SH-SY5Y	ND	PI3K/Akt; Jak2	↑ HO-1(P)
Romero et al. (2010)	Melatonin	0.3–10 nm	Rotenone	30 μM/10 μM	SH-SY5Y	ND	PKC; PI3K/Akt	ND
Quesada et al. (2009)	MGF24	0.1 μg/ml	Rotenone	100 nM	SH-SY5Y	ND	PKC	↑ HO-1(P)
Cañas et al. (2007)	Chondroitin sulfate	0.3–100 μM	Rotenone/Oligo A	10 μM/1 μM	SH-SY5Y	ND	PKC; PI3K/Akt	↑ HO-1(P)
Egea et al. (2007)	Epibatidine	30 nM–30 μM	Rotenone/Oligo A	30 μM/10 μM	BCC	ND	ERK	↑ HO-1(P)
Wu et al. (2006)	EGCG	50–100 μM	Rotenone	5 μM	Endothelial cells	ND	PI3K/Akt; ERK	↑ HO-1(P + M)
Parada et al. (2015)	Curcumin	10–20 μM	Rotenone/Oligo A	30 μM/10 μM	MGC	ND	ND	↑HO-1(P)
Lin et al. (2014)	Resveratrol	10–20 μM	Rotenone	20 μM	SH-SY5Y	ND	ND	↑HO-1(P)

rhLF, recombinant human lactoferrin; 20C, a bibenzyl compound isolated from *Gastrodia elata*; PDC, primary dopaminergic cells; TMP, tetramethylpyrazine; i.g., intragastrically; MGC, mixed glial cultures; MDC, Mes23.5 dopaminergic cells; Oligo A, oligomycin A; PNU282987, α7 nicotinic acetylcholine receptor (nAChR) agonist; 24-amino acid C-terminal peptide of mechano growth factor; CS, chondroitin sulfate; Epibatidine, nicotinic acetylcholine receptors (nAChR) agonist; BCC, bovine chromaffin cells; EGCG, epigallocatechin-3-gallate; PF/β-Ecd, paeoniflorin/β-ecdysterone; TMP, tetramethylpyrazine; GR, glutathione reductase; P, protein; M, mRNA.

### Neuroprotective Role of the Induction of HO-1 in PD

The list of genes regulated by Nrf2/ARE includes over 250 genes, which encode proteins and enzymes involved in antioxidant defense and detoxification (Cores et al., 2020). These genes include classical phase II detoxification enzymes like NQO1, GSTs, etc., and the enzymes involved in GSH biosynthesis, antioxidant defense (e.g., GSH-Px and HO-1), and inflammation (e.g., COX-2 and HO-1) (van Muiswinkel and Kuiperij, 2005; Tebay et al., 2015).

Heme oxygenase-1 (HO-1), a potent antioxidant enzyme regulated by Nrf2, degrades heme to carbon monoxide, free iron, and biliverdin (Consoli et al., 2021). HO-1 has been found at higher concentrations in serum in patients with PD (Sun et al., 2021). HO-1 participates in neuroprotection against OS-dependent injury and has been speculated as a new therapeutic target for PD (Jazwa and Cuadrado, 2010). Tyrrell and others first revealed the cytoprotective effect of HO-1, demonstrating that induction of HO-1 expression mediates an adaptive cytoprotective response to OS in cultured human fibroblasts (Vile et al., 1994; Reeve and Tyrrell, 1999). Particularly interesting is the role played by HO-1 in PD (Schipper et al., 2019). HO-1 induction has been seen to implicate a neuroprotective role on exposure to a variety of PD-associated neurotoxins, both in animal models and in tissue culture (Kwon et al., 2019; Inose et al., 2020). Pharmacological induction of HO-1 by administration of bioactive compounds can exert therapeutic effects against 6-OHDA (Table 7), MPP^+^ (Table 8), MPTP (Table 9), paraquat (Table 10), and rotenone-induced (Table 11) neurotoxicity *in vitro* or *in vivo* PD models.

**TABLE 7:** Summary of the experimental studies involving HO-1 inducer against 6-OHDA-induced PD models.

References	Compound	Compound dose	Toxin	Toxin dose	Model	HO-1 protein	HO-1 mRNA	Signaling	Nrf2
Ji et al. (2021)	Piperlongumine	5–20 µM	6-OHDA	150 µM	PC12	↑	ND	ND	+
Chen et al. (2020)	T-006	3–30 µM	6-OHDA	30 µM	DA neuron	↑	ND	Akt/GSK3β	+
Zhang et al. (2021)	Ginnalin A	10–20 µM	6-OHDA	100 µM	SH-SY5Y	↑	↑	ND	+
Sano et al. (2021)	Fluprostenol	100–500 mM	6-OHDA	50 µM	SH-SY5Y	ND	↑	ERK	+
Ma et al. (2020)	Isoorientin	5–20 µM	6-OHDA	300 µM	SH-SY5Y	↑	ND	AMPK and PI3K/AKT	+
Kwon et al. (2019)	Hyperoside	0.5–2 µM	6-OHDA	200 µM	SH-SY5Y	↑	↑	ND	+
Sheng et al. (2021)	SDA	20–30%	6-OHDA	60 µM	PC12	↑	ND	ND	+
Wu et al. (2021)	Fucoxanthin	1–5 µM	6-OHDA	250 µM	PC12	↑	ND	ND	↓Nrf2-Keap1 binding
Kwon et al. (2019)	Hyperoside	0.5–2 µM	6-OHDA	200 µM	SH-SY5Y	↑	↑	ND	+
Ren et al. (2019)	Tricetin	20–80 µM	6-OHDA	200 µM	SH-SY5Y	↑	ND	ND	+
Funakohi-Tago et al. (2018)	Hydroxytyrosol butyrate	5–10 µM	6-OHDA	100 µM	SH-SY5Y	↑	↑	ND	+
Hou et al. (2018)	Honokiol	5–10 µM	6-OHDA	200 µM	PC12	↑	ND	ND	+
Lee et al. (2018)	Sesquiterpenoid	5–10 µM	6-OHDA	250 µM	PC12	↑	↑	ND	+
Tong et al. (2018)	Simvastatin	1 µM	6-OHDA	100 µM	SH-SY5Y	↑	ND	ND	ND
Moon et al. (2018)	Carbon monoxide	100 µM	6-OHDA	150 µM	C6 glioma cells	↑	↑	ND	+
Izumi et al. (2018)	TPNA10168	10 µM	6-OHDA	250 µM	PC12	↑	↑	Akt	+
Inoue et al. (2018)	HPO-DAEE	10 µM	6-OHDA	70 µM	SH-SY5Y	↑	↑	ND	+
Kao et al. (2017)	1T3O	0.001∼1 µM	6-OHDA	20 µM	SH-SY5Y	↑	↑	Akt	+
Baluchnejadmojarad et al. (2017)	Ellagic acid	50 mg/kg	6-OHDA	2.5 μg/μM	Wistar rats	↑(ELISA)		ND	↑(ELISA)
Masaki et al. (2017)	DDC	1 nmol	6-OHDA	3 μg	C57BL/6N male mice	↑	ND	ND	ND
Kim et al. (2017)	Capillarisin	10–50 µM	6-OHDA	150 µM	SH-SY5Y	↑	↑	JNK	+
Tseng et al. (2016)	DFC	5–10 µM	6-OHDA	100 µM	SH-SY5Y	↑	ND	Akt	ND
Tseng et al. (2016)	DMA	5–10 µM	6-OHDA	100 µM	SH-SY5Y	↑	ND	Akt	ND
Jing et al. (2016)	Tanshinone I	2.5–5 µM	6-OHDA	100 µM	SH-SY5Y	↑	ND	ND	+
Yang et al. (2015)	PACA	5–50 µM	6-OHDA	200 µM	PC12	↑	ND	ND	+
Zhang X. S. et al. (2015)	Tanshinone IIA	5–80 μg/ml	6-OHDA	100 µM	SH-SY5Y	↑	↑	ND	+
Zhang X. S. et al. (2015)	PCA	0.5–1 µM	6-OHDA	100 µM	PC12	↑	↑	ND	+
Zhang Z. et al. (2015)	Chrysin	12 µM	6-OHDA	100 µM	PC12	↑	↑	ND	+
Jin et al. (2015)	Pinocembrin	5–25 µM	6-OHDA	50 µM	SH-SY5Y	↑	ND	ND	+
Zhang et al. (2014)	Urate	200–400 µM	6-OHDA	50 µM	SH-SY5Y	↑	↑	ND	+
Wang X. L. et al. (2014)	Carvedilol	10–20 µM	6-OHDA	100 µM	PC12	↑	↑	Akt	+
Gunjima et al. (2014)	DBL	10–20 µM	6-OHDA	30 µM	SH-SY5Y	↑	↑	PI3K/Akt	+
Li et al. (2013)	Puerarin	10–40 mg/kg	6-OHDA	2.0 g/L	Wistar rats	ND	↑	ND	ND
Chong et al. (2013)	Danshensu	200–400 µM	6-OHDA	250 µM	PC12	↑	ND	PI3K/Akt	+
Bae et al. (2013)	Berberine	10 µM	6-OHDA	60 µM	SH-SY5Y	↑	ND	PI3K/Akt; p38	+
Zhang X. L. et al. (2017)	Berberine	0.25–2 µM	6-OHDA	250 µM	PC12	↑	ND	PI3K/Akt	+
Lin et al. (2012)	Desipramine	10–20 µM	6-OHDA	50 µM	MDC	↑	↑	ERK; JNK	+
Oh et al. (2013)	SRE	10–50 µM	6-OHDA	200 µM	SH-SY5Y	↑	ND	ND	+
Lu et al. (2013)	Resistin	5–10 ng/ml	6-OHDA	75 µM	MDC	↑	ND	ND	ND
Izumi et al. (2012)	DDC	3–30 µM	6-OHDA	200 µM	PC12	↑	ND	PI3K/Akt; p38	+
Kim S. S. et al. (2012)	IGF-1	1–100 nM	6-OHDA	25 µM	PC12	↑	ND	ND	+
Kim Y. et al. (2012)	Licochalcone E	5 µM	6-OHDA	100 µM	SH-SY5Y	↑	↑	ND	+
Deng et al. (2012b)	Sulforaphane	5 µM	6-OHDA	80 µM	PC12	↑	ND	PI3K/Akt	+
Tseng et al. (2012)	Paeonol	0.75–1.5 µM	6-OHDA	40 µM	PCN	↑	ND	ND	ND
Quesada et al. (2011)	MGF24	0.1 μg/ml	6-OHDA	100 µM	SH-SY5Y	↑	ND	PKC	ND
Hara et al. (2011)	Thapsigargin	0.3–30 µM	6-OHDA	80 µM	SH-SY5Y	ND	↑	ND	+
Yamamoto et al. (2010)	Lactacystin	0.3–1 µM	6-OHDA	50 µM	PMC	↑	↑	ND	ND
Yamamoto et al. (2010)	MG-132	30–100 nM	6-OHDA	50 µM	PMC	↑	↑	ND	ND
Yamamoto et al. (2010)	PSI	3–10 nM	6-OHDA	50 µM	PMC	↑	↑	ND	ND
Hwang and Jeong (2010)	Ginsenoside Rb1	30–100 μg/ml	6-OHDA	50 µM	SH-SY5Y	↑	↑	PI3K/Akt	+
Quesada et al. (2009)	MGF24	5–10 μg/ml	6-OHDA	100 µM	SH-SY5Y	↑	ND	Akt	ND
Hwang and Jeong (2008)	kahweol	5–10 μM	6-OHDA	50 µM	SH-SY5Y	↑	↑	PI3K; p38	+
Hwang et al. (2008)	Metallothionein-III	25–50 ng/ml	6-OHDA	50 µM	SH-SY5Y	↑	↑	PI3K; ERK	+
Li et al. (2007)	tBHQ	40 μM	6-OHDA	100 µM	PC12	↑	↑	ND	+
Lee et al. (2006)	Ondamtanggamibang	800 μg/ml	6-OHDA	100 µM	PC12	↑	ND	ND	ND
Lin et al. (2007)	GLNVA	10–100 µM	6-OHDA	100 µM	SH-SY5Y	↑	ND	ND	ND
Hara et al. (2006)	Apomorphine	20–30 µM	6-OHDA	50 µM	SH-SY5Y	ND	↑	ND	+
Muñoz et al. (2004)	N-acetylcysteine	240 mM	6-OHDA	3 μg/μM	Rat	↑	ND	ND	ND
Salinas et al. (2003)	NGF	20 ng/ml	6-OHDA	40 µM	PC12	↑	↑	ND	ND
Zhang et al. (2012)	Baicalein	50–200 μM	6-OHDA	100 µM	PC12	↑	↑	PKCα; PI3K/AKT	+
Wu C. R. et al. (2018)	*Davallia mariesii*	10–250 μg/ml	6-OHDA	50 µM	B35 cells	↑	ND	PI3K/AKT/GSK-3β	ND
Kurauchi et al. (2012)	CAPE	10–30 mg/kg	6-OHDA	2 μg/μM	Mouse	↑	ND	p38 MAPK	+
Hu et al. (2014)	Luteolin	20 μM	6-OHDA	100 µM	PC12	ND	↑	ND	+
Shih et al. (2011)	SHXT	50–200 μg/ml	6-OHDA	100 µM	SH-SY5Y	↑	ND	ND	ND
Kuo et al. (2014)	DFE	25–250 μg/ml	6-OHDA	50 µM	BNC	↑	ND	PI3K/Akt	ND
Park et al. (2014)	α-Iso-cubebene	20–80 μM	6-OHDA	200 µM	SH-SY5Y	↑	ND	PKA/PKB/CREB	ND
Kim et al. (2015)	DHC	0.4–10 μM	6-OHDA	100 µM	SH-SY5Y	↑	ND	ND	+
Luo et al. (2017)	L-F001	1–10 μM	6-OHDA	200 µM	PC12	↑	ND	Akt/GSK-3beta	+

HPO-DAEE, 4-hydroperoxy-2-decenoic acid ethyl ester; 1T3O, 1-tosylpentan-3-one; DDC, 2′,3′-dihydroxy-4′,6′-dimethoxychalcone; DFC, deferricoprogen; DMA. PACA, dimerumic acid, N-propargyl caffeate amide; PCA, protocatechuic acid; DBL, 3,4-dihydroxybenzalacetone; MDC, Mes23.5 dopaminergic cells; SRE, Sanguisorbae Radix extract; IGF-1, insulin-like growth factor -1; PCN, primary cortical neuron cultures; MGF24, 24-amino acid C-terminal peptide of mechano growth factor; lactacystin, a proteasome inhibitor; PMC, primary mesencephalic cultures; PSI, benzyloxycarbonyl-Ile-Glu(O-t-butyl)-Ala-leucinal; MG-132, benzyloxycarbonyl-Leu-Leu-leucinal; tBHQ, tert-butylhydroquinone; GLNVA, glyceryl nonivamide; NGF, nerve growth factor; CAPE, caffeic acid phenethyl ester; SHXT, San-Huang-Xie-Xin-Tang; BNC, B35 neuroblastoma cells; DFE, *Drynaria fortunei* extract; DHC, 5,7-dihydroxychromone; P, protein; M, mRNA.

**TABLE 8:** Summary of the experimental studies involving HO-1 inducer against MPP^+^-induced PD models.

References	Compound	Compound dose	Toxin	Toxin dose	Model	HO-1 protein	HO-1 mRNA	Signaling	Nrf2
Wang T. et al. (2020)	Ghrelin	1–10 µM	MPP^+^	1 mM	SH-SY5Y	↑	ND	ERK1/2	+
Zheng et al. (2020)	CBR-470-1	10 µM	MPP^+^	3 mM	SH-SY5Y	↑	↑	ND	+
Li C. H. et al. (2020)	Ferulic acid	3–10 µM	MPP^+^	250 µM	SH-SY5Y	↑	↑	ERK1/2	+
Zhu J. L. et al. (2019)	SC79	10 µM	MPP^+^	3 mM	SH-SY5Y	↑	↑	Akt	+
Dong et al. (2020)	Thymoquinone	0.5–0.75 µM	MPP^+^	1 mM	SH-SY5Y	↑	ND	ND	+
Bao et al. (2019)	Sulforaphane	1–10 µM	MPP^+^	500 µM	PC12	↑	ND	ND	+
Yan et al. (2018)	Simvastatin	1–1.5 µM	MPP^+^	100 µM	SH-SY5Y	↑	ND	ERK1/2	+
Li C. et al. (2018)	Pinostrobin	1–25 µM	MPP^+^	150 µM	SH-SY5Y	↑	ND	PI3K/AKT; ERK	+
Li M. et al. (2018)	FG-4592	50 µM	MPP^+^	350 µM	SH-SY5Y	↑	ND	ND	+
Wang H. et al. (2018)	BCP	1–2.5 µM	MPP+	50 µM	SH-SY5Y	↑	ND	JNK	ND
Jiang et al. (2014)	Gastrodin	1–25 µM	MPP^+^	100 µM	SH-SY5Y	↑	↑	P38MAPK	+
Jo et al. (2018)	Gintonin	50–100 mg/kg	MPP^+^	250 µM	SH-SY5Y	↑	ND	ND	+
Lee J. A. et al. (2016)	PLE	20–200 μg/ml	MPP^+^	100–200 µM	SH-SY5Y	ND	↑	ND	ND
Liu et al. (2017)	MT-20R	10–100 µM	MPP^+^	150 µM	CGNs	↑	ND	AKT	+
Zou Y. M. et al. (2015)	β-Ecdysterone	1–100 µM	MPP^+^	500 µM	PC12	↑	ND	PI3K	ND
Ye et al. (2012)	Astaxanthin	5–20 µM	MPP^+^	500 µM	PC12	↑	ND	ND	+
Chen et al. (2012)	β-PGG	20–100 µM	MPP^+^	500 µM	PC12	↑	↑	AKT; ERK	+
Moreira et al. (2017)	TUDCA	100 µM	MPP^+^	100 µM	SH-SY5Y	↑	ND	ND	+
Tran et al. (2017)	Amitriptyline	5 µM	MPP^+^	50–200 µM	NNCs	↑	↑	ERK	ND
More and Choi (2017a)	Atractylenolide-I	5–25 µM	MPP^+^	200 µM	SH-SY5Y	↑	↑	ND	ND
Wang et al. (2016)	Pinocembrin	10–30 µM	MPP^+^	200 µM	SH-SY5Y	↑	↑	ERK	+
Huang and Chuang (2010), Chuang et al. (2015)	FGF-9	10–100 ng/ml	MPP^+^	100 µM	PCN	↑	↑	AKT; ERK	+
Cheng et al. (2014)	Edaravone	50–100 µM	MPP^+^	100 µM	PC12	↑	ND	ND	ND
Quesada et al. (2009)	MGF24	0.1 μg/ml	MPP^+^	500 µM	SH-SY5Y	↑	ND	PKC	ND
Wruck et al. (2007)	Luteolin	20 μM	MPP^+^	100 µM	PC12	ND	↑	ERK	+
Liu et al. (2018)	MANF	400 ng/ml	MPP^+^	200 µM	SH-SY5Y	ND	↑	ND	ND
Tiwari et al. (2013)	PLGA	50–400 µM	MPP^+^	100 µM	DNC	↑	ND	ND	ND

CBR-470-1, PGK1 inhibitor; FG-4592, prolyl hydroxylase inhibitor; BCP, β-caryophyllene; PLE, *Paeonia lactiflora* extract; CGNs, cerebellar granule neurons; β-PGG, 1,2,3,4,6-penta-O-galloyl-β-D-glucose; TUDCA, tauroursodeoxycholic acid; NNCs, neocortical neuronal cells; FGF-9, fibroblast growth factor 9; PCN, primary cortical neuron cultures; MGF24, 24-amino acid C-terminal peptide of mechano growth factor; MANF, mesencephalic astrocyte-derived neurotrophic factor; PLE, *Paeonia lactiflora* extract; DNC, dopaminergic neuron cultures.

**TABLE 9:** Summary of the experimental studies involving HO-1 inducer against MPTP-induced PD models.

References	Compound	Compound dose	Toxin	Toxin dose	Model	HO-1 protein	HO-1 mRNA	Signaling	Nrf2
Sheng et al. (2021)	SDA	100–900 mg/kg, p.o. × 4 weeks	MPTP	30 mg/kg/day i.p. for 5 days	Male C57BL/6J mice	↑	ND	ND	+
Dong et al. (2020)	Thymoquinone	10 mg/kg/day, i.p. × 7 d	MPTP	25 mg/kg/day, i.p. × 5 days	C57/BL6 mice	↑	ND	ND	+
Huang et al. (2021)	PSP	30 mg/kg/day, p.o. × 4 weeks	MPTP	30 mg/kg/day, i.p. × 5 days	Male C57BL/6J mice	↑	ND	Akt	+
Choi et al. (2021)	Vinyl sulfones 9d	30 mg/kg/day, p.o. × 3 d	MPTP	20 mg/kg, i.p. four times at 2 h intervals	Male C57BL/6 mice	↑	↑	ND	+
Mohamed et al. (2021)	Tiron	140 and 280 mg/kg, i.p. × 10 days starting 5 days before MPTP injection	MPTP	30 mg/kg/day, i.p. × 5 days	Male albino mice	↑HO-1(ICH)	ND	ND	+
Lin O. et al. (2020)	Trehalose	2% in drinking water	MPTP	30 mg/kg/day, i.p. × 15 times	Male C57BL/6 mice	↑	ND	ND	+
Chen et al. (2020)	T-006	3–10 mg/kg/day, p.o. × 14 d	MPTP	30 mg/kg/day, i.p. × 5 d	Female C57BL/6 mice	↑	ND	Akt/GSK3β	+
Lee J. E. et al. (2020)	KKC080106	30 mg/kg, tid, p.o.	MPTP	20 mg/kg, i.p. four times at 2 h intervals	Male C57BL/6 mice	↑	↑	ND	+
Wang T. et al. (2020)	Piperine analogues-3b	50–100 mg/kg/day, p.o. × 7 d	MPTP	25 mg/kg/day, i.p. × 7 d	Male C57BL/6 mice	↑	ND	ND	+
Li et al. (2020b)	Ferulic acid	50 mg/kg/day, p.o. × 15 d	MPP^+^	25 mg/kg/day, i.p. × 5 d	C57BL/6J mice	↑	↑	ERK1/2	+
Kim et al. (2020)	KKPA4026	30 mg/kg/day, p.o. × 3 d	MPTP	20 mg/kg, i.p. four times at 2 h intervals	Male C57BL/6 mice	↑	ND	ND	+
Qu et al. (2019)	Rosmarinic acid	20 mg/kg, i.g.	MPTP	30 mg/kg, i.p.	C57BL/6 mice	↑	ND	ND	+
Wang Y. et al. (2018)	Pramipexole	0.07–0.15 cm^2^ (TP)	MPTP	30 mg/kg, i.p.	C57BL/6 mice	↑	ND	ND	+
Li M. et al. (2018)	Pinostrobin	0.2–125 µM	MPTP	360 μM	Zebrafish	↑	ND	PI3K/AKT; ERK	+
Li X. et al. (2018)	FG-4592	10 mg/kg/day, i.p.	MPTP	30 mg/kg/day, i.p.	C57BL/6 mice	↑	ND	ND	+
Begum M and Sen (2018)	SNC-80	10 mg/kg	MPTP	30 mg/kg/day, i.p.	Swiss albino mice	↑	ND	ND	+
Jo et al. (2018)	Gintonin	50–100 mg/kg	MPTP	30 mg/kg/day, i.p. × 5 d	C57BL/6N mice	↑	ND	ND	+
Kabel et al. (2018)	Linagliptin	3–10 mg/kg/day	MPTP	*	80 Balb/c mice	↑(ELISA)		ND	+(ELISA)
Huang et al. (2017)	Uric acid	25 mg/kg/day × 13 d	MPTP	25 mg/kg/day, i.p. × 7 d	C57BL/6 mice	ND	↑	ND	+
Moreira et al. (2017)	TUDCA	50 mg/kg × 3 d	MPTP	40 mg/kg	C57BL/6 mice	↑	ND	ND	+
More and Choi (2017b)	Atractylenolide-I	30 mg/kg/10 ml	MPTP	10 mg/kg/10 ml	C57BL6/J mice	↑	↑	PI3K/AKT	ND
Liu et al. (2017)	MT-20R	60–180 mg/kg × 7 d	MPTP	30 mg/kg/day, i.p. × 5 d	C57BL/6 Mouse	↑	ND	AKT	+
Luo et al. (2017)	L-F001	35–70 mg/kg × 7 d	MPTP	40 mg/kg	C57BL/6 mice	↑	ND	Akt/GSK-3beta	+
Ozkan et al. (2016)	DHA	36 mg/kg/day	MPTP	20 mg/kg	C57BL/6 mice	↑	ND	ND	+
Lee J. A. et al. (2016)	ITC-57	30 mg/kg × 3 d	MPTP	20 mg/kg/2 h, i.p. × 4	C57BL/6J mice	↑	↑	ND	+
Lee J. A. et al. (2015)	VSC2	10 mg/kg/day × 3 d	MPTP	20 mg/kg, i.p. × 4	C57BL/6 mice	↑	↑	ND	+
Woo et al. (2014)	Vinyl sulfones	10 mg/kg	MPTP	20 mg/kg/2 h, i.p. × 4	C57BL/6 mice	↑	↑	ND	+
García et al. (2014)	S-Allyl cysteine	120 mg/kg, i.p. × 5 d	MPTP	30 mg/kg/day, i.p. × 5 d	C57BL/6 mice	↑	ND	ND	+
Tiwari et al. (2013)	PLGA	1 mg/kg/day, i.p. × 7 d	MPTP	20 mg/kg/2 h, i.p. × 4	Swiss albino mice	↑	ND	ND	ND
Chinta et al. (2012)	DHB	100 mg/kg, i.p.	MPTP	2 × 20 mg/kg, 12 h apart	C57BL/6 mice	↑	ND	p38MAPK; JNK	ND
Jazwa et al. (2011)	Sulforaphane	50 mg/kg	MPTP	30 mg/kg	mice	↑	ND	ND	+
Lee et al. (2009)	DHB	100 mg/kg, i.p.	MPTP	2 × 20 mg/kg, 12 h apart	C57BL/6 mice	↑	ND	ND	+HIF

TP, transdermal patch; SNC-80, DOR agonist; L-F001, a multifunction ROCK inhibitor; DHA, docosahexaenoic acid; ITC-57, novel synthetic isothiocyanate; VSC2, (E)-1-(2-((2-methoxyphenyl)sulfonyl)vinyl)-2-chlorobenzene); PLGA, poly(lactic-co-glycolic) acid; DHB, the prolyl hydroxylase inhibitor 3,4-dihydroxybenzoate; HIF, hypoxia-inducible factor.

**TABLE 10:** Summary of the experimental studies involving HO-1 inducer against paraquat-induced PD models.

References	Compound	Compound dose	Toxin	Toxin dose	Model	HO-1 protein	HO-1 mRNA	Signaling	Nrf2
de Oliveira et al. (2017a) (2018a)	Carnosic acid	1 μM	Paraquat	100 μM	SH-SY5Y	ND	ND	ND	+
Rasheed et al. (2020)	Resveratrol		Paraquat		mouse	↑	ND	ND	+
Li et al. (2012)	tBHQ	Oral feeding	Paraquat	7 mg/kg	C57BL/6 mice	↑	ND	ND	+
Li et al. (2012)	tBHQ	40 μM	Paraquat	100–300 μM	PC12	↑	ND	ND	+
de Oliveira et al. (2018b)	Naringenin	80 μM	Paraquat	100 μM	SH-SY5Y	ND	ND	ND	+
Alural et al. (2015)	Lithium	2–5 mM	Paraquat	0.5 mM	SH-SY5Y	ND	↑	ND	+
de Rus Jacquet et al. (2017)	Allium sativum	1–10 μg/ml	Paraquat	2.5 μM	PMC	↑	↑	ND	+
de Rus Jacquet et al. (2017)	Trifolium pratense	1–10 μg/ml	Paraquat	2.5 μM	PMC	↑	↑	ND	+
de Rus Jacquet et al. (2017)	Amelanchier arborea	1–10 μg/ml	Paraquat	2.5 μM	PMC	↑	↑	ND	+

6-HITC, 6-(methylsulfinyl)hexyl isothiocyanate, which is a naturally occurring isothiocyanate; tBHQ, tert-butylhydroquinone; PMC, primary midbrain cultures.

**TABLE 11:** Summary of the experimental studies involving HO-1 inducer against rotenone-induced PD models.

References	Compound	Compound dose	Toxin	Toxin dose	Model	HO-1 protein	HO-1 mRNA	Signaling	Nrf2
Terada et al. (2020)	Ziprasidone	0.1–1 µM	Rotenone	1 µM	PC12	ND	↑	ND	+
Duvigneau et al. (2020)	Cannabidiol	10 µM	Rotenone	80 nM	PMC	ND	↑	ND	ND
Lin O. et al. (2020)	Monascin	100–400 mg/kg/day, p.o., for 28 days	Rotenone	2–3 mg/kg, i.p. for 42 days	Male SD rats	↑	ND	ND	+
Arab et al. (2021)	Dapagliflozin	1 mg/kg//kg/2day, p.o., over 3 weeks	Rotenone	1.5 mg/kg, s.c., every other day over 3 weeks	Adult male Wistar rats	↑Activities of HO-1	ND	ND	+
Wei et al. (2020)	Ellagic acid	100 mg/kg/days, p.o. × 35 d	Rotenone	1 mg/kg, s.c. 6 times a week for consecutive 5 weeks	C57BL/6J male mice	↑	ND	ND	+
El-Ghaiesh et al. (2020)	Metformin	100 or 200 mg/kg, every 24 ± 2 h, volume = 4 ml/kg	Rotenone	1 mg/kg, s.c. every 48 h, volume = 4 ml/kg	Male Swiss albino mice	↑	ND	ND	+
Wang T. et al. (2020)	Danshensu	15–60 mg/kg, p.o. × 15 d	Rotenone	30 mg/kg	Male C57BL/6 mice	↑	ND	PI3K/AKT	+
Zhu L. et al. (2019)	SC79	10 µM	Rotenone	300 nM	SH-SY5Y	↑	↑	Akt	+
de Rus Jacquet et al. (2017)	Allium sativum	1–10 μg/ml	Rotenone	20 nM	PMC	↑	↑	ND	+
de Rus Jacquet et al. (2017)	Trifolium pratense	1–10 μg/ml	Rotenone	20 nM	PMC	↑	↑	ND	+
de Rus Jacquet et al. (2017)	Amelanchier arborea	1–10 μg/ml	Rotenone	20 nM	PMC	↑	↑	ND	+
Liu et al. (2016)	PF/β-Ecd	4–3.2 μM–/0.4–3.2 μM	Rotenone	1 μM × 24 h	PC12	↑	↑	Akt	+
Michel et al. (2017)	TTMP	2 mg/kg, i.p. × 4 w	Rotenone	2 mg/kg, s.c. × 4 w	SD rat	↑	ND	ND	+
Cui et al. (2016)	Curcumin	100 mg/kg, bid, i.g. × 50 d	Rotenone	1 ml/kg/d, bid, i.g. × 50 d	Lewis rats	↑	ND	Akt	+
Minelli et al. (2009)	Cyclo (His-Pro)	50 μM	Rotenone	100 μM	PC12	↑	ND	p38 MAPK	+
Zakharova et al. (2018)	rhLF	25 mg/kg	Rotenone	2.75 mg/kg	Wistar rats	+	↑	↑HO-1(M)	+
Engel et al. (2018)	Duloxetine	2–5 μM	Rotenone	10 μM	SH-SY5Y	+	↑	PI3K/Akt	+
Zhang C. et al. (2017)	20C	1–10 μM	Rotenone	4 μM	SH-SY5Y	↑	ND	PI3K/Akt	+
Zhang C. et al. (2017)	20C	1–10 μM	Rotenone	4 μM	PC12	↑	ND	PI3K/Akt; GSK3β	+
Zakharova et al. (2018)	rhLF	25 mg/kg	Rotenone	2.75 mg/kg	Wistar rats	ND	↑	ND	+
Engel et al. (2018)	Duloxetine	2–5 μM	Rotenone	10 μM	SH-SY5Y	ND	↑	PI3K/Akt	+
Zhang X. L. et al. (2017)	20C	1–10 μM	Rotenone	4 μM	SH-SY5Y	↑	ND	PI3K/Akt	+
Zhang X. L. et al. (2017)	20C	1–10 μM	Rotenone	4 μM	PC12	↑	ND	PI3K/Akt; GSK3β	+
Pan et al. (2016)	Safranal	10–50 μg/ml	Rotenone	100 nM	PDC	↑	↑	ND	+
Zhou et al. (2016)	Sulforaphane	50 mg/kg	Rotenone	30 mg/kg	C57BL/6 mice	↑	ND	ND	+
Huang et al. (2016)	20C	0.01–1 μM	Rotenone	4 μM	PC12	↑	↑	ND	+
Cui et al. (2016)	Curcumin	100 mg/kg, bid × 50 d	Rotenone	1 mg/kg/d, bid × 46 d	Lewis rats	↑	ND	Akt	+
Parada et al. (2015)	Curcumin	10–20 μM	Rotenone/Oligo A	30 μM/10 μM	MGC	↑	ND	ND	ND
Lin et al. (2014)	Resveratrol	10–20 μM	Rotenone	20 μM	SH-SY5Y	↑	ND	ND	ND
Lin et al. (2012)	Desipramine	10–20 μM	Rotenone	3 μM	MDC	↑	↑	ERK; JNK	+
Dal-Cim et al. (2012)	Guanosine	1 mM	Rotenone/Oligo A	30 μM/10 μM	SH-SY5Y	↑	ND	PI3K/Akt; GSK-3β	ND
Parada et al. (2010)	PNU282987	1–10 μM	Rotenone/Oligo A	30 μM/10 μM	SH-SY5Y	↑	ND	PI3K/Akt; Jak2	ND
Romero et al. (2010)	Melatonin	0.3–10 nm	Rotenone	30 μM/10 μM	SH-SY5Y	ND	ND	PKC; PI3K/Akt	ND
Quesada et al. (2009)	MGF24	0.1 μg/ml	Rotenone	100 nM	SH-SY5Y	↑	ND	PKC	ND
Cañas et al. (2007)	CS	0.3–100 μM	Rotenone/Oligo A	10 μM/1 μM	SH-SY5Y	↑	ND	PKC; PI3K/Akt	ND
Egea et al. (2007)	Epibatidine	30 nM–30 μM	Rotenone/Oligo A	30 μM/10 μM	BCC	↑	ND	ERK	ND
Wu et al. (2006)	EGCG	50–100 μM	Rotenone	5 μM	Endothelial cells	↑	↑	PI3K/Akt; ERK	ND
Jo et al. (2018)	Gintonin	50–100 mg/kg	Rotenone	200–500 nM	SH-SY5Y	↑	ND	ND	+

rhLF, recombinant human lactoferrin; 20C, a bibenzyl compound isolated from *Gastrodia elata*; PDC, primary dopaminergic cells; TMP, tetramethylpyrazine; i.g., intragastrically; MGC, mixed glial cultures; MDC, Mes23.5 dopaminergic cells; Oligo A, oligomycin A; PNU282987, α7 nicotinic acetylcholine receptor (nAChR) agonist; MGF24, 24-amino acid C-terminal peptide of mechano growth factor; CS, chondroitin sulfate; Epibatidine, nicotinic acetylcholine receptors (nAChR) agonist; BCC, bovine chromaffin cells; EGCG, epigallocatechin-3-gallate.

## Future Perspectives

In the last decade, many research groups have developed induced pluripotent stem cell-based protocols to generate three-dimensional, multicellular, neural organoids to study the pathophysiology of PD (Lázaro et al., 2017; Rai and Singh, 2020; Costamagna et al., 2021; Outeiro et al., 2021). Organoids provide almost full features of PD pathology and physiology. The main advantage of using organoids as a PD model is that it shows very close association with *in vivo* conditions; thus, organoids are very easy to recapitulate all the features of PD. As compared to the conventional two-dimensional culture model, these new three-dimensional organoids provide new hope for drug screening. Recently, Outeiro and others developed microfluidic platforms to investigate specific molecular mechanisms associated with PD (Fernandes et al., 2016). Microfluidic platforms have shown PD-relevant phenotypes, including ROS production and mitochondrial dysfunction. Fernandes et al. designed a microfluidic device to understand their cell-cell and biochemical communication. The connected chambers allowed rapid diffusion of molecules from one camber to another. The device was integrated with pneumatic valves, which helped in controlling the fluid routing and cellular microenvironment and simulating the paracrine signaling. The authors studied the spreading of α-Syn and mutual communication between different cell types (neurons and glia). They observed diffusion of ROS from a chamber containing activated microglia to the other chamber that contained healthy neuroglioma cells indicating the role of ROS for neuronal functional impairment (Fernandes et al., 2016). The microfluidic device was used to study the transport of mitochondria along dopaminergic axons isolated from mice (Lu et al., 2012). A recent study used a microfluidic platform to dissect the mitochondrial dysfunctions associated with a genetic form of PD with dynamin-related GTPase optic atrophy type 1 (OPA1) mutations (Iannielli et al., 2019), revealing that axons of OPA1 mutant dopaminergic neurons exhibit a significant reduction of mitochondrial mass. This defect causes a significant loss of dopaminergic synapses, which worsens in long-term cultures. Therefore, PD-associated depletion of mitochondria at synapses might precede loss of neuronal connectivity and neurodegeneration. Seidi and others used microfluidic platforms to study the effect of 6-OHDA that induces neuronal apoptosis in PC12 cells. This represented an *in vitro* model of PD, which revealed that low and high concentrations of 6-ODHA decreased the viability of neuronal cells due to apoptosis and necrosis, respectively. Thus, these concentration gradient studies were considered as useful information for creating an *in vitro* model of PD to induce the highest level of apoptosis in cells (Seidi et al., 2011). They may provide a useful approach for generating *in vitro* models of disease for drug discovery applications.

Microfluidics is a rising star in the development of innovative approaches in drug discovery and screening, particularly in screening natural product drugs based on chemical properties, pharmacological effects, and drug cytotoxicity. But in the present stage, these newly developed *in vitro* models of PD and microfluidic platforms were not used to study the effect of the Nrf2/HO-1 activator (Lee J. A. et al., 2020). Future research is expected to elucidate the detailed molecular mechanism of Nrf2/HO-1 activator which regulates Nrf2 activation and HO-1 induction in these newly developed *in vitro* models of PD leading to the development of novel drugs that target Nrf2/ARE/HO-1.

## Conclusion

**FIGURE 7:**
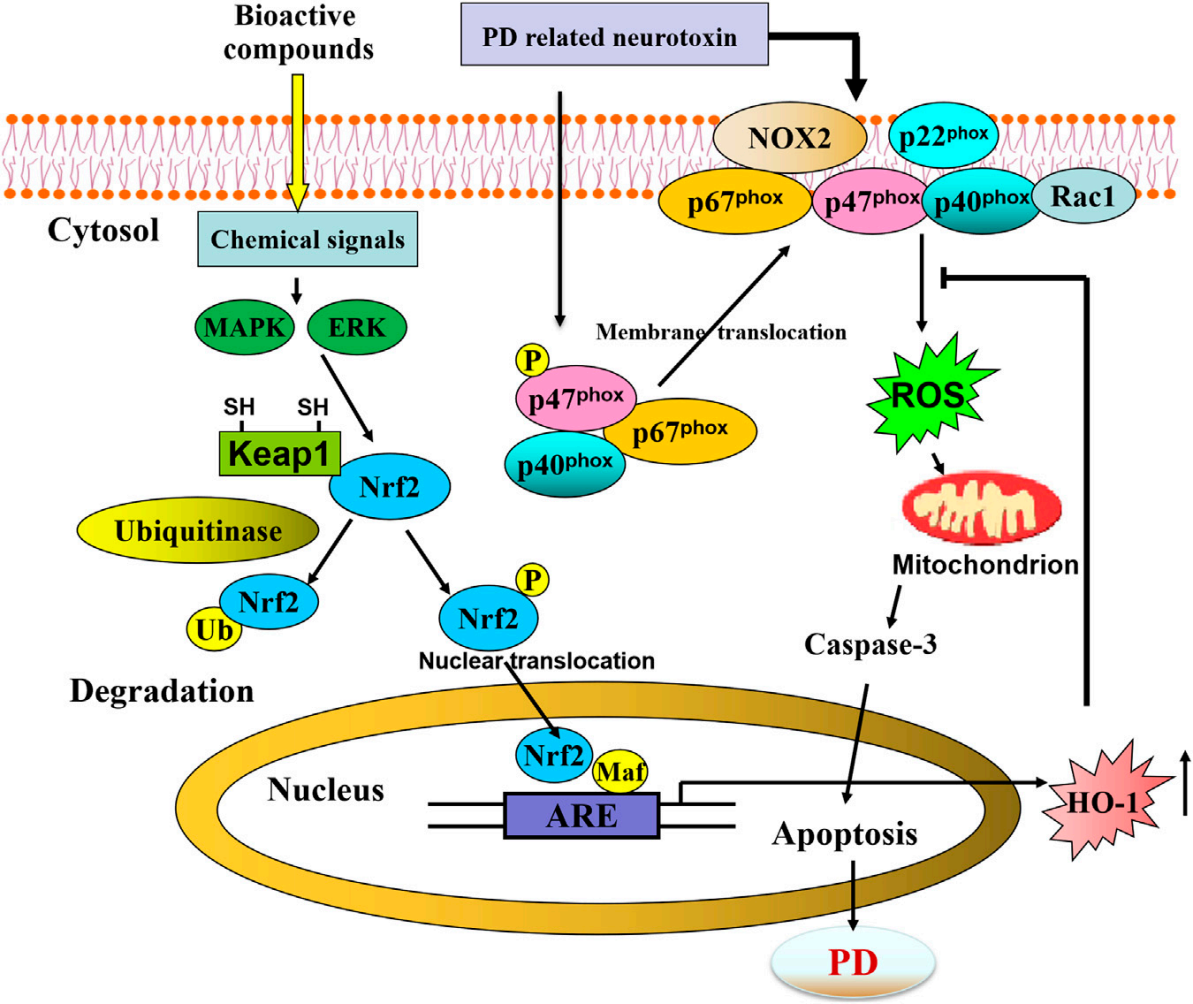
Schematic representation of bioactive compounds-mediated neuroprotective against PD through activating Nrf2/ARE/HO-1 pathway.

Emerging evidence has suggested that the Nrf2/ARE pathway plays a crucial role in cellular adaption by controlling orchestrated cytoprotective proteins, including HO-1, to counteract OS in PD, thereby providing a promising optimal therapeutic target against PD (Figure 7). By using various PD-related neurotoxin-induced *in vitro* and *in vivo* models, recent preclinical studies provide direct compelling evidence that the contribution of the pharmacological modulation of the Nrf2/ARE/HO-1 pathway exerts neuroprotection in PD.
